# Establishing a Consensus-Based Framework for the Use of Wearable Activity Trackers in Health Care: Delphi Study

**DOI:** 10.2196/55254

**Published:** 2024-08-23

**Authors:** Kimberley Szeto, John Arnold, Erin Marie Horsfall, Madeline Sarro, Anthony Hewitt, Carol Maher

**Affiliations:** 1 Alliance for Research in Exercise, Nutrition and Activity, Allied Health and Human Perfomance University of South Australia Adelaide Australia; 2 Allied Health and Human Perfomance University of South Australia Adelaide Australia; 3 Southern Adelaide Local Health Network South Australia Health Adelaide Australia

**Keywords:** wearable activity tracker, health care, physical activity, sedentary behavior, wearable, wearables, wearable tracker, tracker, wearable technology, support, exercise, prevention, management, monitor, promote, survey, utility

## Abstract

**Background:**

Physical activity (PA) plays a crucial role in health care, providing benefits in the prevention and management of many noncommunicable diseases. Wearable activity trackers (WATs) provide an opportunity to monitor and promote PA in various health care settings.

**Objective:**

This study aimed to develop a consensus-based framework for the optimal use of WATs in health care.

**Methods:**

A 4-round Delphi survey was conducted, involving a panel (n=58) of health care professionals, health service managers, and researchers. Round 1 used open-response questions to identify overarching themes. Rounds 2 and 3 used 9-point Likert scales to refine participants’ opinions and establish consensus on key factors related to WAT use in health care, including metrics, device characteristics, clinical populations and settings, and software considerations. Round 3 also explored barriers and mitigating strategies to WAT use in clinical settings. Insights from Rounds 1-3 informed a draft checklist designed to guide a systematic approach to WAT adoption in health care. In Round 4, participants evaluated the draft checklist’s clarity, utility, and appropriateness.

**Results:**

Participation rates for rounds 1 to 4 were 76% (n=44), 74% (n=43), 74% (n=43), and 66% (n=38), respectively. The study found a strong interest in using WATs across diverse clinical populations and settings. Key metrics (step count, minutes of PA, and sedentary time), device characteristics (eg, easy to charge, comfortable, waterproof, simple data access, and easy to navigate and interpret data), and software characteristics (eg, remote and wireless data access, access to multiple patients’ data) were identified. Various barriers to WAT adoption were highlighted, including device-related, patient-related, clinician-related, and system-level issues. The findings culminated in a 12-item draft checklist for using WATs in health care, with all 12 items endorsed for their utility, clarity, and appropriateness in Round 4.

**Conclusions:**

This study underscores the potential of WATs in enhancing patient care across a broad spectrum of health care settings. While the benefits of WATs are evident, successful integration requires addressing several challenges, from technological developments to patient education and clinician training. Collaboration between WAT manufacturers, researchers, and health care professionals will be pivotal for implementing WATs in the health care sector.

## Introduction

### Background

Physical activity (PA) is critical in preventing and managing many noncommunicable diseases [1]. For chronic disease populations, PA can mitigate disease progression, improve physical function, and reduce the risk and burden of comorbid health conditions [2]. In the hospital setting, extremely low levels of patient PA are linked to poor outcomes, including functional decline, increased frailty and disability, and increased mortality risk [3-5]. Inactivity is also associated with higher rates of hospitalization [6-8], longer length of hospital stay [9,10], and increased risk of readmission [11,12]. Global guidelines recommend adults perform at least 150 minutes of moderate or 75 minutes of vigorous-intensity PA weekly, muscle strengthening exercise at least 2 days per week, and reduce sedentary behavior (SB) [2]. Yet just 1 in 5 adults globally meet these guidelines [13], resulting in a substantial global burden of physical inactivity, contributing to millions of premature deaths each year [1], and costing an estimated US $53.6 billion annually in health care costs and loss of productivity [14].

The need to address PA in the health care sector is clear. Indeed, organizations such as the World Health Organization [15] and the International Society for Physical Activity [16] have identified health care as a crucial setting for investment and implementation of strategies to promote PA. Health care professionals (HCPs) are strategically positioned to influence a wide range of people either living with or at risk of developing many diseases that could be alleviated or prevented through increased PA. Accordingly, PA has been endorsed as a “vital sign” that should be routinely assessed by HCPs to identify inactive individuals, prompt interventions targeted at increasing PA, and form a baseline for such interventions [17-19].

Wearable activity trackers (WATs) offer substantial potential for measuring and influencing patient PA. They have gained widespread acceptance among researchers, HCPs, and the general population. Consumer-oriented WATs, such as Fitbits, use accelerometers to measure PA, and have seen an uptick in popularity [20]. These consumer-oriented WATs may also incorporate additional sensors, like heart rate and blood oxygen saturation monitors, allowing users to track multiple health parameters via the device interface and associated smartphone applications. Compared with other methods of PA assessment, such as self-report questionnaires, WATs demonstrate superior validity, reliability, and reduced biases [21,22]. Furthermore, there is a substantial body of evidence demonstrating the effectiveness of WAT-based interventions for augmenting PA across diverse populations, with subsequent benefits like improved physical function, body composition, and blood pressure [23-25].

Despite considerable evidence demonstrating the opportunities and value of using WATs in health care settings, their routine use in health care has not yet been achieved. Nonetheless, HCPs and researchers are endeavoring to adopt WATs and are exhibiting optimism about their potential for PA measurement, informing exercise prescription and PA promotion, and fostering patient motivation and self-monitoring [26,27]. While smaller-scale efforts that have been made so far are encouraging and demonstrate interest from a spectrum of stakeholders, barriers to broader and routine use of WATs in health care persist. These barriers range from practical obstacles related to the WAT itself, such as battery life and wear issues, to clinician workload, software and data access limitations, lack of interdisciplinary support, and costs and resource concerns [26,28,29]. This highlights the need for standardized and coordinated approaches to WAT deployment in health care. A more systematic approach could facilitate the comparison and amalgamation of data across similar populations from disparate settings, circumvent recurring issues, and enhance the desired outcomes of using WATs in health care.

### Objectives

This study aimed to establish a standardized and consensus-based framework for the quality use of WATs in health care settings. To do this, we sought to identify the most important metrics and device characteristics for health care–focused WAT use, identify suitable clinical populations and settings for WAT deployment, and explore stakeholders’ perceptions of the most significant barriers to WAT adoption in health care, alongside potential solutions.

## Methods

### Study Design

A 4-round web-based Delphi study was conducted to meet the study aims between March 2021 and June 2022. This research adhered to the Conducting and Reporting of Delphi Studies (CREDES; Multimedia Appendix 1 [30]).

### Ethical Considerations

This research was conducted in accordance with the Helsinki declaration of 1975, as revised in 2000. The University of South Australia’s Human Research Ethics Committee granted ethical approval (approval number: 203069). Given the web-based survey design, opening the survey weblink and completing the survey were considered implied informed consent. Participants could elect to withdraw from this study if requested.

### Participants and Recruitment

We recruited participants representing three stakeholder groups with experience or expertise on the topic: (1) HCPs, (2) researchers, (3) health service managers (eg, department officials or heads of services), or any combination thereof. Potential participants were identified through multiple channels: HCPs from a preceding study [26], professional associations and networks, participant referrals (ie, invitees were encouraged to share with relevant contacts), by searching for health service managers and officials from health networks (eg, government health departments), and international researchers with relevant expertise. Potential participants were emailed study information and invited to participate. A follow-up email was sent after 1 week to nonrespondents. We aimed to recruit 50 participants based on recommendations for Delphi studies with multiple stakeholder groups [31]. We anticipated similar representation from HCPs and researchers, with fewer health service managers (approximately 15% of the total sample) due to the limited number of these positions. All participants who agreed were invited to complete all survey rounds, regardless of earlier rounds’ completion.

### Delphi Surveys

Four survey rounds were distributed via email (SurveyMonkey). Surveys were piloted by 5 people who were not participants (2 HCPs, 1 researcher, and 2 both HCP and researcher) to ensure clarity, timeliness, and appropriate topic exploration, with amendments made if required. Surveys took 15-25 minutes to complete, and participants had 3 weeks to complete each survey. Reminders were sent after 7 and 14 days. Results from each round were provided to participants within subsequent rounds, with summaries incorporated within the surveys for context.

### Round 1

The Round 1 survey (Multimedia Appendix 2) aimed to generate items and identify broad themes on the topic. Round 1 collected data on participants’ professional experience with WATs, and used 12 open-ended questions to explore perspectives on the following: clinical value of WATs, suitable clinical populations and settings, important metrics, important device characteristics, and barriers and enablers to use in health care settings. At the outset of the survey, we defined “wearable activity trackers” as “a wearable device for tracking activity-related metrics such as steps, sleep, energy expenditure (eg, calories), and in some cases, activity minutes.” Open-response questions were analyzed thematically, categorized, and converted to items for the subsequent round.

### Round 2

The Round 2 survey (Multimedia Appendix 3) used findings from Round 1 to refine opinions and establish consensus on 64 items across 4 sections and introduced a fifth section on software, based on its emergence as a significant topic in Round 1. The first 4 sections considered key metrics, essential WAT characteristics, and usefulness for various populations and settings. Participants rated their agreement with items on a 9-point Likert scale (eg, “it is critically important for the wearable to measure ‘x’”: 1=strongly disagree to 9=strongly agree), and were encouraged to provide comments on open-response questions in each section. One multiple-choice question determined participants’ preferred wear site for WATs. The fifth section on software comprised multiple-choice, item rating, and open-response questions which were tailored for participants with direct WAT experience in health care.

### Round 3

The Round 3 survey (Multimedia Appendix 4) comprised 3 sections and was designed using insights from earlier rounds. It sought to refine opinions and reach consensus on new items, rerate items that did not meet consensus in Round 2, and further explore barriers and potential strategies identified in Round 1. The first section required participants to rate 28 items on the same topics explored in Round 1 (6 rerating, 22 new). The second section explored software, requiring participants to rate 11 new items, and included an optional open-response question for additional comments. The third section explored barriers, and required participants to select 1 multiple-choice response for what they considered to be the most important barrier to using WATs in health care across 3 categories (patient, clinician and interdisciplinary team, and health care–system), and included open-response questions to provide suggestions for strategies to address the barriers. Barriers were explored in Round 3, given the length and item volume of the Round 2 survey.

### Round 4

Between Rounds 3 and 4, three authors (KS, CM, and JA) drafted a 12-item checklist containing key elements for clinicians and service planners to consider when developing procedures for using WATs in health care settings (Multimedia Appendix 5 [26-28,32-47]). Results from Rounds 1-3 and the Consolidated Framework for Implementation Research (CFIR) [48] informed the constituents of the draft checklist. The CFIR provides a structure for approaching and evaluating intervention implementation and recognizes that to be effective, new innovations need to be adapted to “fit” the needs of a setting while retaining their “core” components. The draft checklist included “core” elements to consider when developing procedures for using WATs in different settings while allowing the user to adapt details based on their specific circumstances. Each item included prompts and explanations. During Round 4, participants evaluated the checklist items’ clarity and usefulness and provided overall feedback.

### Data Analysis

Response rates, participant characteristics, and responses on Likert scales and multiple-choice questions were analyzed using descriptive statistics (frequency of responses and percentages). Results were presented for each stakeholder type (“health system” and “research”) and for the entire sample. Qualitative data from open-response questions were analyzed thematically and organized into emergent categories and themes, or converted to items for rating in subsequent rounds.

### Consensus Agreement

Consensus was determined based on responses on 9-point Likert scales. Participants rated their agreement with statements regarding either the importance, appropriateness, or usefulness of items. Ratings were categorized as “not important/appropriate/useful” (1-3), “neutral” (4-6), and “critically important/appropriate/useful” (7-9) based on the 9-point GRADE methodology [49]. Items were scored as either:

- “Critically important/appropriate/useful”: ≥75% of participants rating ≥7, and ≤15% of participants rating ≤3.
- “Somewhat important/appropriate/useful”: 50%-74% rated an item ≥7
- “Not important” when <50% rated an item ≥7.

Item scores were grouped based on responses from different stakeholder groups: “healthcare” (HCPs and health-service officials) and “research” (researchers). Scores were summarized by group and for the entire sample. To establish consensus, we considered item scores from the 2 groups, and scores from the entire sample. Scores from each group were considered, as we were interested in different perspectives that the types of stakeholders may have, and if this influenced the overall rating of items.

Items reached consensus based on the following criteria:

- “Critically important/appropriate/useful”: when this was the score for both groups or for one group only and the overall score was “critically important/appropriate/useful.”
- “Somewhat important/appropriate/useful” when this was the score for both groups or for one group and the overall rating was “somewhat important/appropriate/useful.”
- “Consensus not met”: the score from one group was either “critically important” or “somewhat important,” and the score from the other group was “not important.”
- “Not important” (omitted from exploration in subsequent rounds): when the rating from both groups was “not important.”

## Results

### Participants

**Figure 1:**
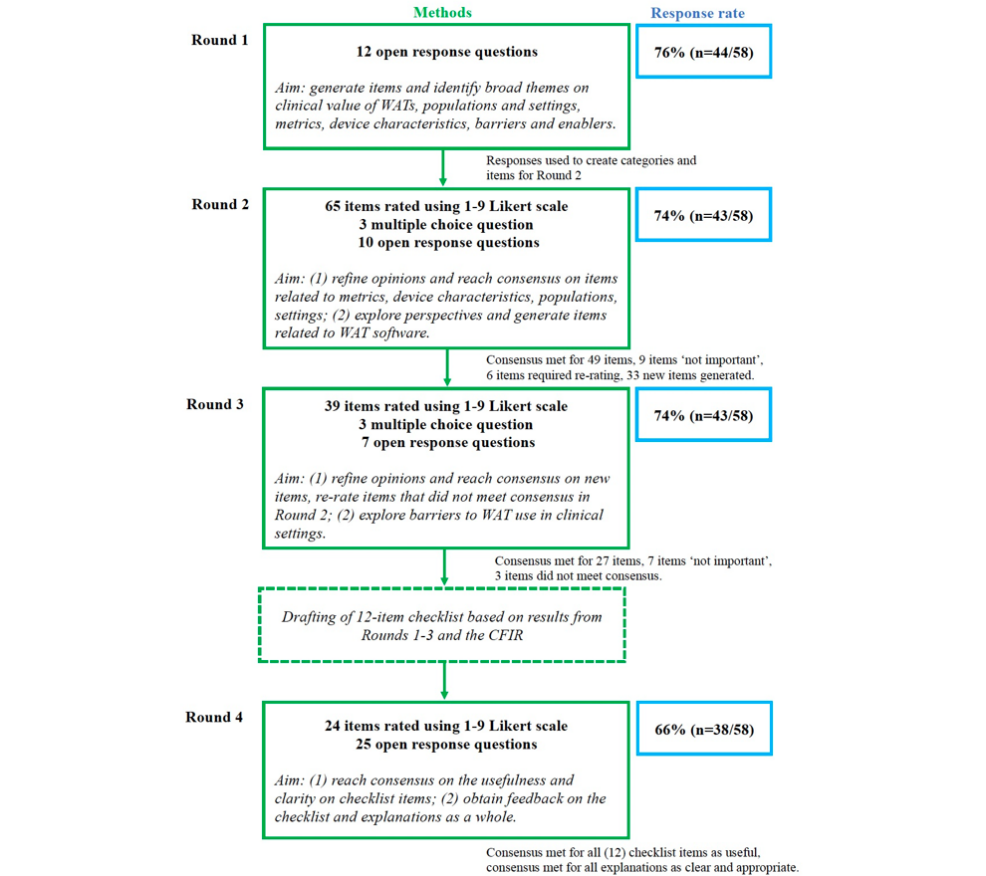
Overview of Delphi process. WAT: wearable activity tracker; CFIR: Consolidated Framework for Implementation Research.

Of 82 potential participants who were directly invited to the study, 38 (46%) individuals expressed interest in participating. An additional 20 individuals contacted the research team after learning about the study via word of mouth, yielding a total sample of 58. Response rates for rounds 1 to 4 were 76% (n=44), 74% (n=43), 74% (n=43), and 66% (n=38) respectively, with 52 (90%) participants responding to at least 1 round, and 28 (48%) participants completing all 4 rounds. An overall summary of the structure of the Delphi rounds, item consensus, and response rates is shown in Figure 1.

### Round 1

Of the 44 participants who completed Round 1, 8 participants were health service managers, 24 participants were HCPs, and 17 participants were researchers (Table 1). All participants considered WATs to be useful in health care settings, predominantly for the purposes of monitoring patient activities (ie, PA, SB, and sleep), intervening in patient PA, and monitoring physiological parameters (ie, heart rate). Participants provided a wide variety of responses for suitable populations and settings (eg, people with metabolic conditions, rehabilitation, inpatient, and outpatient). Various PA and physiological metrics were suggested, with 26 discrete metrics identified. The most frequently reported metrics were daily step count (n=27), daily PA minutes (n=20), and heart rate (n=19). A total of 28 device characteristics were reported as important for clinical settings, which were categorized under battery and charging (eg, long battery life), wear (eg, waterproof), device interface (eg, real-time feedback), data (eg, easy data access), and other (eg, affordable cost). Qualitative analysis suggested that the selection of metrics and characteristics would be influenced by the purpose of use, the population and setting, and patient factors such as goals and level of mobility. Numerous barriers and enablers to using WATs in health care settings were reported, which were grouped into 4 categories: device-related (eg, battery: short battery life), patient-related (eg, patient suitability: cognition and mobility), clinician-/multidisciplinary team-related (eg, time constraints and competing demands: setting up devices) and system-level (eg, lack of procedures and systems: distributing and managing devices). Enablers were generally the inverse of barriers (for instance, if short battery life was a barrier, long battery life was an enabler). A full summary of results from Round 1 is available in Multimedia Appendix 2.

**Table 1:** Demographics of the cohort (n=44).

Characteristics			Round 1 responses, n (%)
**Type of participant**			
	Health care professional	24 (55)	
	Health service managers	8 (18)	
	Researcher	17 (39)	
**Country**			
	Australia	41 (94)	
	United States	1 (2)	
	United Kingdom	1 (2)	
	Spain	1 (2)	
**Years of experience in field**			
	Up to 5	3 (7)	
	>5-10	6 (14)	
	>10-20	14 (32)	
	>20	21 (47)	
**Clinical field (** **n=33** **)**			
	Physiotherapy	23 (70)	
	Exercise physiology	2 (5)	
	Other allied health	2 (5)	
	Medical doctor	3 (9)	
	Nurse	1 (3)	
	Other	2 (5)	

### Round 2

Round 2 comprised five sections. The first 4 sections required item rating to indicate agreement with the importance of different metrics and characteristics of WATs for clinical use, and to indicate agreement with the appropriateness of using WATs with different clinical populations and settings.

The consensus was reached for 26/64 items as being “critically important” (metrics: 2/14, device characteristics: 8/19), or “highly appropriate” (populations: 6/10, settings and purposes: 10/21), and 23/64 items as being “somewhat important” (metrics: 2/14, device characteristics: 8/19), or “somewhat appropriate” (populations: 3/10, settings and purposes: 10/21; Tables 2 and 3). Nine items reached an outcome of “not important.” Six items did not reach consensus and were rerated in the following round. Interestingly, despite step count being the most frequently reported useful metric in Round 1, 74% of participants overall rated it as “critically important” in Round 2, leading to an overall score of “somewhat important” (<75% of participants rating ≥7). Given its frequent mention and relevance in research [23,50], the team decided this item warranted rerating in the next round. Most participants (72%) considered the wrist to be the most appropriate wear site, with 18 participants providing open-response comments on the wear site. Comments mostly related to wear site affecting the validity and reliability of data, as well as patient engagement and preferences, with themes identified that wear site should consider both the purpose of using WATs (ie, is patient engagement the priority), and the patient context (ie, walking speed; Multimedia Appendix 3).

**Table 2:** Device-related items that reached consensus in Rounds 2 and 3.

Item				Percentage agreement (≥7 on 9-point Likert scale)		
				Round 2 (n=43)		Round 3 (n=43)
**Metrics**						
	**Critically important (≥75% rated ≥7) ≥7**					
		Daily minutes of PA^a^	95		N/A^b^	
		Daily minutes of SB^c^	81		N/A	
		Daily step count	74		86	
	**Somewhat important (50%-74% rated ≥7)**					
		Intensity of PA (heart rate zones)	56		N/A	
		Details of PA intensities (eg, light/moderate/vigorous)	—^d^		74	
		All daily activities (physical activity, sedentary time, sleep)	58		N/A	
		Sit to stand transitions	—		58	
		Position of SB (eg, sitting/standing/lying down)	—		56	
		Heart rate	44^e^		58^e^	
		On versus off body time (wear time)	—		60	
**Characteristics**						
	**Critically important (≥75% rated ≥7)**					
		Easy to charge	95		N/A	
		Comfortable to wear day and night	93		N/A	
		Water resistant	81		N/A	
		Easy to clean and disinfect	81		N/A	
		Simple data syncing process	98		N/A	
		Simple data download or export process	91		N/A	
		Store at least 5 days of data without syncing or downloading	88		N/A	
		Easy to navigate and set up	93		N/A	
		Provide data that is easy to interpret	—		88	
		Wrist-worn is most appropriate for patient acceptability and compliance	MCQ^f^		77	
		Adaptable wear site for different populations and individuals (eg, those with walking frames)	—		84	
	**Somewhat important (50%-74% rated ≥7)**					
		Battery that lasts at least 2 days on a single charge	74		N/A	
		Battery that lasts at least 5 days on a single charge	63		N/A	
		Quick to charge (eg, reach full charge in 1 hour)	70		N/A	
		Measure steps in slow ambulators	72		N/A	
		Ability to set personalized goals	63		N/A	
		Simple data interface (eg, interpret key data at a glance)	74		N/A	
		Real-time feedback is provided on the interface	67		N/A	
		Data can be accessed remotely by clinician	56		N/A	
		Aesthetically pleasing	51^e^		50	
		Ability to set specific reminders	—		56	
		Ability to select between different metrics for viewing	—		70	
		Ability to wear at different bodily locations	49^e^		63	
		Wear site may need to be adapted for different purposes (eg, assessment vs intervention)	—		56	

^a^PA: physical activity.

^b^N/A: not applicable (agreement met in prior round).

^c^SB: sedentary behavior.

^d^not rated (identified from this round).

^e^Disagreement between groups.

^f^MCQ: multiple choice question.

**Table 3:** Wearable activity tracker usefulness items that reached consensus in Rounds 2 and 3.

Item				Percentage agreement (≥7 on 9-point Likert scale)		
				Round 2 (n=43)		Round 3 (n=43)
**Patient populations**						
	**Highly appropriate (≥75% rated ≥7)**					
		Cardiovascular	95		N/A^a^	
		Pulmonary	88		N/A	
		Metabolic (eg, obesity, bariatrics, and diabetes)	98		N/A	
		Mixed rehabilitation	86		N/A	
		Chronic pain	79		N/A	
		Older adults (geriatrics and aged care)	88		N/A	
	**Somewhat appropriate (50%-74% rated ≥7)**					
		Orthopedic	74		N/A	
		Neurological	74		N/A	
		Pediatric	65		N/A	
		Oncology	—^b^		70	
		Mental Health	—		65	
		Disability sector	—		72	
**Measuring activity**						
	**Highly appropriate (≥75% rated ≥7)**					
		During home-based rehabilitation	95		N/A	
		Following a hospital admission (after discharge)	84		N/A	
		Outpatient settings	88		N/A	
		Community-based settings (inside or outside the home)	95		N/A	
		Residential aged-care (home-based or live-in facilities)	81		N/A	
	**Somewhat appropriate (50%-74% rated ≥7)**					
		Prior to an elective hospital admission	60		N/A	
		During an inpatient hospital admission	70		N/A	
**Measuring physiological parameter**						
	**Highly appropriate (≥75% rated≥7)**					
		During home-based rehabilitation	76		N/A	
	**Somewhat appropriate (50%-74% rated ≥7)**					
		During an inpatient hospital admission	56		N/A	
		Following a hospital admission (after discharge)	70		N/A	
		Outpatient settings	65		N/A	
		Community-based settings (inside or outside the home)	72		N/A	
		Residential aged-care (home-based or live-in facilities)	65		N/A	
		Prior to an elective hospital admission	47^c^		65	
**Intervening on activity**						
	**Highly appropriate (≥75% rated ≥7)**					
		During home-based rehabilitation	86		N/A	
		Outpatient settings	77		N/A	
		Community-based settings (inside or outside the home)	86		N/A	
		Following a hospital admission (after discharge)	77		N/A	
	**Somewhat appropriate (50%-74% rated ≥7)**					
		Prior to an elective hospital admission	63		N/A	
		During an inpatient hospital admission	58		N/A	
		Residential aged-care (home-based or live-in facilities)	72		N/A	

^a^N/A: not applicable (agreement met in prior round).

^b^Not rated (identified from this round).

^c^Disagreement between groups.

Section 5 focused on the software aspects of using WATs in health care settings. Participants with direct experience were specifically asked to rate their satisfaction with the existing WAT software they had used in health care settings. Of the 32 respondents to this item, most (59%) rated their software experience as neutral or dissatisfied. Twenty-nine participants described the software they had used and how they thought software could be improved to better meet the needs of health care settings. The majority reported using proprietary software linked to the device or reading outputs from the device interface, and some either invested in third-party software or developed their own. Those who described difficulties generally reported that software was difficult to use, time-consuming, and expensive (eg, logging in and out of separate accounts to view data on consumer WATs, or needing to purchase specialized software to download data). Participants provided numerous suggestions regarding the requirements of WAT software to meet the needs of health care settings, which were converted into items for consensus rating in Round 3. A full summary of results for Round 2 is available in Multimedia Appendix 3.

### Round 3

Round 3 comprised 3 sections. The first section covered metrics, device characteristics, populations, and settings and purposes, and focused on rating new items and rerating items that did not meet consensus in Round 2. The second section focused on software, and involved rating items generated from Round 2 to identify “ideal” software features. The third section focused on barriers to WAT use in health care.

Consensus was reached for 11/39 items as being “critically important” (metrics: 1/13, device characteristics: 3/11, software: 7/11), and 16/39 items as being “somewhat important” (metrics: 3/13, device characteristics: 4/11, software: 4/11) or “somewhat appropriate” (patient populations: 3/3, settings and purposes: 1/1; Tables 2-4). Four items did not meet consensus (metrics: 2/13, device characteristics: 2/11). Seven items reached an outcome of “not important.”

**Table 4:** Ideal software items that reached consensus in Round 3 (n=43).

Item		Percentage agreement (≥7 on 9-point Likert scale)
**Critically important (≥75% rated ≥7)**		
	Present relevant data at varying levels of simplicity	93
	Centralized access of patient data	84
	Access to multiple patients’ data simultaneously	79
	Wireless data upload or export	93
	Batch data downloads	84
	Access to raw data sets	77
	Ability to conduct separate or additional analyses to those provided by the device	79
**Somewhat important (50%-74% rated ≥7)**		
	Capacity to input self-report data (eg, RPE^a^, pain, and fatigue VAS^b^)	53
	Provide useful and relevant data on the wearable interface (eg, without further analysis needed)	70
	Capacity to download and access data sets instantly	72
	Automatically present data in different formats (eg, graphs, tables, summary scores without having to edit or analyze data sets)	65

^a^RPE: rate of perceived exertion.

^b^VAS: visual analog scale.

The barriers section of the survey asked participants to identify the most significant obstacles to WAT use in health care across 3 categories: patient-related, clinician– and interdisciplinary team–related, and health care system. Results were presented by group (health care and research participants) and overall. No single patient-related barrier dominated, but there were nuances between health care and research participants (Multimedia Appendix 4). Most health care participants (37%) considered “clinical unsuitability” to be the most important, and most research participants (37%) considered “patient reluctance” to be the most important. Participants from both groups considered “time constraints and competing demands” to be the most important clinician-related barrier (overall: 51%, health system: 53%, research: 50%), and “lack of funding and resources” to be the most important health care system–related barrier (overall: 58%, health system: 63%, research: 54%). Participants provided strategies to address these barriers, with the top 5 strategy categories, in order of frequency, being: clinician information and support (19 mentions); standardized approach (17 mentions), broader involvement of teams or families and caregivers (15 mentions), top-down support (14 mentions), and patient education and support and improved funding and resourcing (13 mentions each). A full summary of results for Round 3 is available in Multimedia Appendix 4.

### Round 4

A draft 12-item user checklist was developed based on insights from Rounds 1-3 and guided by the CFIR. This checklist was designed to assist HCPs and health service managers in planning to implement WATs across various health care settings. During Round 4, participants were presented with the draft checklist with the goal of gauging consensus on the usefulness of individual items and assessing the clarity and appropriateness of explanations. There was clear support for individual items and explanations, with every item on the checklist reaching consensus as being “very useful,” and all explanations reached consensus as being “very clear and appropriate” (Multimedia Appendix 5 [26-28,32-47]). Fourteen participants provided comments on the checklist as a whole, which was predominantly positive feedback. A few comments related to the presentation of the final product, shortcomings, and the clarity or amount of information. Twenty-three participants provided feedback on individual items and explanations, which was mostly general comments related to the content of items (eg, suggestions to provide more detail and citations regarding the accuracy and validity of devices in different contexts). Other common responses included suggestions for additional information, suggestions to condense information, specific suggestions on wording or formatting, and feedback on the clarity of wording. All comments for individual items and the expanded checklist including elaboration statements for each of the 12 items are provided in Multimedia Appendix 5 [26-28,32-47].

## Discussion

### Principal Results

This Delphi study brought together HCPs, health service managers, and researchers to gather expert opinions regarding the application of WATs in health care settings. Findings revealed that participants believed that WATs offer utility for monitoring and improving patient PA in a wide variety of clinical populations and health care settings. The study identified various metrics and device characteristics as critical, with the specific choice of metrics being contingent on the clinical context and patient factors. Software was acknowledged as a vital element, with most participants being unsatisfied with the currently available software. Key barriers to WAT adoption in health care were recognized in relation to patients, clinicians, and health care system categories, with time constraints and resource limitations being major obstacles. To overcome these, participants suggested strategies such as clinician support, standardized approaches, and improved funding. The study culminated in the development of a 12-item checklist to assist HCPs and service planners in successfully integrating WATs within various health care settings.

### Potential for Widespread Adoption of WATs in Health Care

Our findings highlight widespread interest in using WATs in a very wide range of clinical populations, settings, and purposes. Various clinical groups were identified as suitable for WAT applications, across different health care contexts. This multifaceted application of WATs is corroborated by existing literature, with previous studies using WATs with post-surgical patients and acutely ill patients in hospitals for daily step count monitoring to predict hospital readmission and length of stay [10,11], as well as using WATs in various community-dwelling chronic disease populations [24,51,52] and various hospitalized populations [25] for promoting patient PA. Together, these findings highlight the versatility of using WATs in health care settings and their promising role in advancing patient care.

### Toward More Systematic Integration of WATs in Health Care

This study identified a desire for more standardization in WAT use in health care, yet with such a range of different potential applications for WATs, a universal approach seems unfeasible. Nevertheless, there was consensus on essential components for diverse WAT applications. Out of 45 important elements identified, 21 were classified as “critically important,” encompassing 3 metrics, 11 device characteristics, and 7 software characteristics. Furthermore, an additional 24 elements were classified as “somewhat important.” Recognizing these pivotal components can steer HCPs and potential users to select devices and software that meets their clinical needs. These elements were incorporated into the user-oriented Wearable Activity Tracker Checklist for Health care (WATCH) developed from this study, which is presented in the companion article [53]. Given that the barriers this study identified were similar to those described in international studies [28,29], it seems likely that the core elements we identified will be relevant for applications in different locations.

### Strengths and Limitations

This study is the first to use an expert consensus approach to advance the use of WATs in health care settings. It adhered to rigorous Delphi methodology, including defining consensus, having clear criteria for accepting, rerating, and omitting items, defining the number of rounds to be performed a priori and piloting surveys before sending them to participants [54]. The average response rate of 73% was another strength of this study, given the large number of items and larger size of the panel, both of which have been associated with lower response rates for Delphi studies [55].

This study has several limitations that warrant consideration. First, a limitation of the Delphi methodology is that it uses a limited sample of participants to represent various stakeholder groups of interest. It is possible that the participants in this Delphi study may not represent the broader population of HCPs, health service managers, and researchers with expertise and experience in the application of WATs in health care. While participants in this Delphi study represented various professions and had diverse experiences with WATs in different populations and settings, the sample was predominantly Australian. This may limit the generalizability of our findings to other contexts, as digital technology and data landscapes vary significantly across countries. These variations can influence the accessibility, usability, and integration of wearable technologies and associated software in health care practices. For instance, differences in regulatory environments, data privacy laws, and technological infrastructure can impact the deployment and effectiveness of WATs. Therefore, HCPs from different jurisdictions may hold different attitudes and perspectives. A further limitation of the Delphi methodology is that, as a consensus-driven approach, it can potentially overlook important opinions or knowledge if they are not widely held by the sample [31]. In this study, some metrics were considered more important than others for clinical use. However, the combination of multiple metrics (and metrics considered “less important”) should not be overlooked in gaining other important insights from WAT data. For example, heart rate data can be used to determine if devices are being worn by patients. This Delphi study did not involve patients, who are also end users of WATs, thereby lacking their perspectives. Patients will be an important stakeholder group in future research as the integration of WATs in health care becomes more widespread. Finally, as WAT technology continues to advance rapidly, the considerations and factors explored in this study may require updating.

### Implications

Our study identified various barriers to the use of WATs in health care that will need to be addressed to support widespread implementation. First, patient-related barriers included clinical unsuitability for certain conditions, and patient reluctance, often rooted in apprehension or lack of understanding. Addressing this will require comprehensive patient education and selecting WATs suitable for specific patient needs. Additionally, clinician-related barriers like time constraints necessitate streamlining of WAT integration processes. Incorporating training programs for HCPs is likely to enhance their competency in efficiently using WATs. At the system level, financial constraints and lack of resources appear to be significant obstacles. Strategically advocating for funding, possibly through demonstrating the long-term cost benefits of WATs in patient care, may be instrumental to addressing this. Lastly, interoperability and data integration into electronic health records will be pivotal in the future for clinicians to effectively access and interpret data. The study also reveals a substantial gap between the ideal and actual capabilities of currently available WAT devices and software in health care settings. Current options fall short in areas deemed critical by participants, particularly in software features, wearability, and data accuracy. This highlights an urgent call for the innovation of WAT technology and software tailored specifically for health care applications. Such innovations may include the integration of behavior change and gamification techniques (eg, goal-setting, feedback loops, rewards, and nudges) into devices to foster use and engagement, potentially leading to increased effectiveness of PA promotion efforts. A collaborative approach between WAT manufacturers, researchers, and HCPs will be imperative to develop solutions that not only address the practical necessities of health care settings but also rigorously uphold data privacy standards required for handling patient information.

### Conclusions

This Delphi study offers valuable insights into the prospects and challenges of integrating WATs in health care settings for PA promotion. The collective perspectives of HCPs, health service managers, and researchers underscore the broad potential of WATs across diverse clinical scenarios. Yet, for WATs to be fully effective in patient care, several hurdles must be addressed, ranging from patient education to technological advancements specific to the health care sector. The evolving nature of WAT technology will necessitate continuous collaboration and re-evaluation. As the health care landscape seeks more personalized and data-driven approaches, the integration of WATs presents a promising avenue to enhance PA promotion and patient outcomes, optimize clinical processes, and elevate the overall quality of health care delivery.

## Appendix

Multimedia Appendix 1 CREDES checklist.

Multimedia Appendix 2 Round 1 survey and results.

Multimedia Appendix 3 Round 2 survey and results.

Multimedia Appendix 4 Round 3 survey and results.

Multimedia Appendix 5 Round 4 survey and results.

## Abbreviations

CFIR: Consolidated Framework for Implementation Research
CREDES: Conducting and Reporting of Delphi Studies
HCP: health care professional
PA: physical activity
SB: sedentary behavior
WAT: wearable activity tracker
WATCH: Wearable Activity Tracker Checklist for Health Care
