# Supplementary material for: Establishing a Consensus-Based Framework for the Use of Wearable Activity Trackers in Health Care: Delphi Study
Source: JMIR Mhealth Uhealth. 2024 Aug 23;12:e55254. doi: 10.2196/55254 (PMC11380062; doi:10.2196/55254)
Supplement: Multimedia Appendix 1 [file mhealth_v12i1e55254_app1.docx]

**CREDES checklist items, taken from Table 5. Selection of experts, definition of consensus and quality of reporting [1]**

| **Transparency and quality of reporting** | | |
| --- | --- | --- |
| **Item** | **Manuscript Section** | **Page No.** |
| Purpose well defined | Background | 4-6 |
| Rationale for Delphi Selection of experts clearly justified | Methods | 6-7 |
| Clear description of methods | Methods | 6-11 |
| Flow chart | Methods | 13, Figure 1 |
| Clear definition of consensus | Methods | 10-11 |
| Pilot test of instruments | Methods | 7 |
| Data analysis clearly justified and reported | Methods | 10 |
| Information of rounds | Methods | 7-10 |
| Transparent reporting of results | Results | 11-20 |
| Discussion of limitations | Discussion | 21-22 |
| Adequacy of conclusions | Conclusions | 23-24 |

| **Selection criteria expert panel** |  |
| --- | --- |
| Member of organisation |  |
| Recognised authority | 🗸 |
| Relevant clinical academic expertise | 🗸 |
| Geographical scope | 🗸 |
| Setting/work field | 🗸 |
| Profession/ stakeholder | 🗸 |

1. Jünger, S., S.A. Payne, J. Brine, L. Radbruch, and S.G. Brearley, *Guidance on Conducting and REporting DElphi Studies (CREDES) in palliative care: Recommendations based on a methodological systematic review.* Palliative medicine, 2017. **31**(8): p. 684-706.
