# Supplementary material for: Establishing a Consensus-Based Framework for the Use of Wearable Activity Trackers in Health Care: Delphi Study
Source: JMIR Mhealth Uhealth. 2024 Aug 23;12:e55254. doi: 10.2196/55254 (PMC11380062; doi:10.2196/55254)
Supplement: Multimedia Appendix 2 [file mhealth_v12i1e55254_app2.pdf]

Round 1 Survey and Results summary

Round 1 Survey Questions

Some information about you:

1) What is your current role?
2) If your current role is in administration (i.e. head of department), or research/academia, do you have any former experience as a clinician?
3) Please state the country and state that you are employed in.
4) Please describe your current area of work (e.g. if research - field of interest; if clinician - population/setting etc.)
5) How many years of experience do you have in the field?
6) Do you have any experience with wearable activity monitors as a clinician?
7) Describe your experience with wearable activity monitors in non-clinical settings (e.g. research, or personal use).
8) What clinical populations have you used wearable activity monitors with? (If not applicable, please enter N/A).

Are wearables of use?

1) In your opinion, what can wearable activity monitors be used for in clinical settings?

Which types of patients?

2) In your opinion, to which patient populations are wearables best suited?

Which environments?

3) In your opinion, to which clinical settings are wearables best suited?

Which metrics?

4) In your opinion, which metrics from wearable activity monitors are useful/relevant in clinical settings?

Important Features

5) In your opinion, what features of wearable activity monitors help make them suitable for clinical practice? (this may include, but isn't limited to, practical considerations such as battery life, easy to use software, wear site on body etc.)

Barriers to Use

6) Within clinical settings what are 3 (or more) patient-related barriers to the use of wearable activity monitors?
7) Within clinical settings, what are 3 (or more) clinician-related barriers to the use of wearable activity monitors?
8) Within clinical settings what are 3 (or more) healthcare system-level barriers to the use of wearable activity monitors?

Enablers to Use

9) Within clinical settings, what are 3 (or more) patient-related factors that help enable or support the use of wearable activity monitors?
10) Within clinical settings, what are 3 (or more) clinician-related factors that help enable or support the use of wearable activity monitors?
11) Within clinical settings, what are 3 (or more) healthcare system-level factors that help enable or support the use of wearable activity monitors?

Final comments

12) Is there anything else you would like to add?

Round 1 Results Summary

Demographic information

A total of n=44 participants took part in the Round 1 Survey, and came from clinician, health system administration and academic backgrounds (Table 1). Many fell into more than one category. Most participants came from the field of physiotherapy, with other disciplines representing exercise physiologists, medical doctors, nurses and allied health professionals. Approximately half of all participants had over 20 years of experience in their respective fields. Approximately half of participants were based in South Australia, while others came from other Australian states, and three from international countries.

**Table 1. Demographics of cohort**	
	**Number of participants (*n=44*)**
**Type of participant:**	
Clinician	24
Health system administration	8
Academic	17
**Clinical field (*n=33*):**	
Physiotherapy	23
Exercise physiology	2
Other allied health	2
Medical doctor	3
Nurse	1
Other	2
**Location:**	
SA	24
Australia (not SA)	17
Overseas	3
**Years of experience:**	
Up to 5	3
>5-10	6
>10-20	14
>20	21

Table 2 displays the professional fields of experience and expertise that participants came from. Participants represented a wide range of fields. The specific clinical populations that the cohort have worked with was broad, and largely encompassed various non-communicable and lifestyle disease populations (predominantly neurological, cardiovascular, and metabolic), paediatrics, geriatrics and older adults, with some healthy/sub-clinical (e.g., office workers) and athletic representation. Similarly, the clinical settings that participants have experience was broad, and included various hospital, community-based and aged-care settings. Participants who came from academic backgrounds represented a range of areas of research, with digital health and informatics being common, along with various areas of health and physical activity research.

**Table 2. Professional fields represented across cohort**		
		**Frequency**
**Clinical Populations**	Cardiovascular	10
	Neurological	10
	Metabolic	7
	Geriatrics and Older adults	6
	Pulmonary	5
	Orthopaedic	5

	Paediatrics (including infants)	5
	Obesity	4
	Amputees	3
	Oncology	3
	Office workers	2
	Disability	1
	Sporting and Athletic populations	1
**Clinical Settings**	Private practice allied health	6
	Mixed rehabilitation	5
	General rehabilitation	4
	Surgical	4
	Medical	3
	Acute care and ICU (hospital inpatients)	3
	Sub-acute care (hospital)	3
	Home rehabilitation	3
	Education	3
	Telehealth	1
	Palliative care	1
**Research fields of interest**	Digital health and Informatics	8
	Physical activity and Sedentary behaviour	3
	Chronic disease management	3
	Behaviour change and Activity promotion	3
	Sports participation	3
	Rehabilitation	3
	Exercise and Nutrition interventions (including technology-based interventions)	2
	Healthy aging	2
	Chronic pain	2
	Models and Standards of care in allied health	2
	Falls	1
	Running	1
	Time-use epidemiology	1
	Weight loss	1

Experience with WAMs and interest in this area

Approximately half of participants had experience using WAMs with patients in a clinical role, and half of the participating academics have used them in clinical research. The clinical populations and settings, and research applications that WAMs have been used in largely reflected the professional backgrounds of the cohort. The vast majority who didn't have personal experience using WAMs in clinical practice or research had experience using them personally. All considered WAMs to be of value in clinical practice.

Relevant populations and clinical settings for use

The diversity of the cohort produced a wide variety of responses and insight across the areas of interest. It became apparent that WAMs hold potential for use in most clinical populations and across a variety of settings. Some participants provided interesting and pertinent information related to their specific areas of expertise/experience (such as cognition, mobility etc.). A common or specific patient population or clinical setting or metric did not emerge as most suitable to use WAMs with. Appropriate clinical judgement was considered an important factor, regardless of the specific population or setting suggested, with many noting that clinical judgement would be required to ensure individual patient safety and appropriateness (e.g. ability to independently mobilise, cognitively able to manage device use).

Patient populations

Many participants indicated that any and all medically stable patient populations for whom increased activity was indicated were suitable, and fewer suggestions were made for specific clinical populations. More suggestions for patient factors – as opposed to specific clinical cohorts –

were made, and included things like ability to independently mobilise, being motivated by and interested in objective data, and familiarity with technology. Where specific patient populations were suggested, this largely reflected the professional fields of individual participants.

Clinical settings

Similarly, the clinical settings suggested were highly varied, and reflected participants' respective experience and areas of expertise. Broadly, this included inpatient and outpatient hospital settings, community-based settings, and aged care. It was considered by many that WAMs could be useful in settings with less face-to-face contact (such as outpatients, home and community settings) as well as those with more face-to face contact (such as inpatient rehab and aged care).

Relevant metrics

It was widely considered that the type of metric used would depend on the patient, their goals, and the setting, though steps and daily activity minutes (including physical activity, sedentary time and sleep) were most frequently reported. Many reported heart rate data, with other vital signs (such as oxygen saturation and blood pressure) reported to a lesser extent. Body position (such as sitting/lying down etc.), distance and GPS data, and speed were also noted. A full summary of relevant metrics reported is displayed in Table 3.

Table 3. Relevant Metrics

**Metric**	**Frequency**
Daily step count	27
Daily (moderate-vigorous) physical activity minutes	20
Heart rate	19
Distance	11
Sleep time and/or patterns	11
Resting/sedentary/inactive time	8
Positional/postural change (lie-to-sit, sit-to-stand, etc.) (including gyros – angular velocity sensors)	7
Exercise/physical activity intensity or effort	7
24hr pattern/mapping of activity (PA, sedentary time & sleep)	6
Any or All	6
Standing/Upright time	4
GPS/location tracking – time spent in each location	4
Speed/pace (m/s)	3
Blood pressure	3
Oxygen saturation (Sp02)	2
Swimming distance/activity in water	2
Energy expenditure	2
Recovery time – e.g. EPOC	1
Elevation	1
METS – base/resting metabolic rate	1
Centre of gravity motion	1
Respiratory/breathing rate	1
Electroencephalography during sleep	1
Stress levels	1
Arm/leg swings	1
Blood glucose levels	1

Important device features

Many device features were listed as being important (Table 4), and were mostly related to battery, wear, data, and device interface. The most frequently reported features were: having an intuitive interface, long battery life, and easily accessed, exported and downloaded data. Other features included: waterproofing, wearability and the devices' ability to provide instantaneous data to wearers. Other considerations relating to hygiene, instructions for use, device availability and compatibility were mentioned. Less commonly noted were sound features, branding and data security.

Table 4: Important Device Features

		Frequency
**Battery & Charging**	Long battery life:	21
	12hrs	2
	24hrs	2
	8 days for full week cycle	1
	Easy and quick to charge	4
**Wear**	Waterproof	10
	Comfort / Wearability:	9
	Easily attached & worn	6
	Non-irritable band & material	2
	Wear site:	8
	Arm / Wrist band	11
	Thigh	2
	Waist / Pants	2
	Ankle	1
	T-shirt	1
	Shoe insoles	1
	Ability to alter or adjust wear site	7
	Size of device – dimensions & weight suitable	5
	Aesthetic & social acceptability	4
	Hygiene / Infection control – easy to clean & disinfect	4
	Durable device and materials – e.g. not easily damaged	3
	Practical and functional	2
	Brand of device	1
**Interface**	Instant or real-time feedback of data to patient	10
	Remote monitoring of device/s – e.g. from the clinic or home	5
	Ability to set & modify goals – with prompts/reminders	3
	Ability to track/measure performance & achievement	2
	Display of data on device – graphics	2
	Compatibility – data, across devices, operating systems	2
	Sound & volumes – adequate and adjustable	1
**Data**	Ability to easily access, download, export, analyze & share data	18
	Accurate, reliable and valid data	3
	Sufficient data storage and long-term tracking	2
	Shock resistant / Sensitive to movement	2
	Data security	1
**Other Considerations**	Affordable / cost effective	7
	Device availability	3
	Use without supporting materials or devices – stand-alone	2
	Instructions & support – readily available	1

Barriers and enablers

A wide range of barriers and enablers to effective use of WAMs were reported across categories, and device-related barriers emerged as an additional category. In many cases, the enablers were the inverse of the barriers, though some unique enablers emerged.

Barriers

Device-related barriers were related to battery, software and interface, wear, costs and privacy. Frequently reported barriers included short battery and charging issues, difficulty navigating device interfaces, hard to understand data and outcomes, uncomfortable to wear, and high costs to invest in devices. Full summary of device-related barriers in Table 5.

**Table 5. Device-related barriers**	
Battery	Short battery life; difficulty charging
Software and interface	Difficult to use and navigate; difficult to understand data and outcomes; poor sensitivity and accuracy; unclear validity and reliability; poor connectivity and internet; slow logging in/out and syncing
Wear	Uncomfortable and irritating; not waterproof; cleaning requirements; bulky; sizing issues for different size patients; unattractive
Costs	Expensive to purchase; ongoing maintenance; too much variety; high rate of change/becoming 'old' quickly
Privacy	Tracking; confidentiality; data ownership

Patient-related barriers were related to suitability (of patient), willingness (of patient), support and accessibility, and knowledge and education. Most frequently reported barriers included low motivation and interest, forgetting to wear, lack of resources/funding to obtain own device, poor digital literacy and older age, poor health literacy, impaired cognition, and personal concerns about privacy and safety. Full summary of patient-related barriers in Table 6.

**Table 6. Patient-related barriers**	
Suitability	Impaired cognition; reduced physical capacity and limited mobility; physical size of patient (device needs to fit)
Willingness	Low motivation and interest; poor compliance (e.g., not wearing devices, losing/damaging devices; compliance with therapy); personal concerns (e.g., privacy, safety)
Support and accessibility	Lack of resources to obtain own device; inappropriate internet and technological support; lack of family/carer support; reliance on and limited time with clinician
Knowledge and education	Poor health literacy; lack of understanding purpose of use; poor digital literacy (often accompanying older age)

Clinician and interdisciplinary team-related barriers related to time constraints and competing demands, lack of skills and training, lack of procedures and systems, lack of team involvement, ethical considerations, lack of funding, individual clinician factors, and macro-level factors. Time constraints and competing demands were reported to impact the ability to perform the tasks related to WAM use (such as set-up, syncing, interpreting data, and cleaning). Lack of skills and training in how to use the devices and perform the required tasks was also frequently reported. Similarly, many reported that there was a lack of procedures and systems in place to inform use and manage the practical aspects of using WAMs in clinical settings. Full summary of clinician and interdisciplinary team-related barriers in Table 7.

Table 7. Clinician and interdisciplinary team-related barriers	
Time constraints and competing demands	Setting up devices; logging in/out and accessing data; downloading and syncing data; analysing and interpreting data; cleaning; reporting relevant information; problem solving for patient
Lack of skills and training	How to use devices and software; interpreting/analysing data and outcomes; simplifying and communicating necessary information and outcomes to patient; integration into current practice
Individual clinician factors	Unwilling to use; poor digital literacy; lack of knowledge and skills
Lack of procedures and systems	How to use devices and software; distribution and managing devices; infection control; charging; data storage; data analysis and interpretation.
Lack of team involvement	Lack of leadership; multi-disciplinary involvement; technological support

Healthcare system-related barriers were related to lack of skills and training, lack of procedures and systems, lack of team involvement, ethical considerations, lack of funding, and macro-level factors. Barriers relating to lack of skills and training, lack of procedures and systems, and lack of team involvement overlapped with clinician and interdisciplinary team-related barriers. Lack of funding was frequently reported as impacting obtaining devices, and supporting ongoing maintenance and resource management. Macro-level barriers relating to unclear cost-benefit, unclear benefits and patient populations, and unclear outcomes and values were reported often. Full summary of healthcare system-related barriers in Table 8.

Table 8. Healthcare system-related barriers	
Lack of skills and training	How to use devices and software; interpreting/analysing data and outcomes; simplifying and communicating necessary information and outcomes to patient; integration into current practice
Lack of procedures and systems	How to use devices and software; distribution and managing devices; infection control; charging; data storage; data analysis and interpretation.
Lack of team involvement	Lack of leadership; multi-disciplinary involvement; technological support
Ethical considerations	Privacy and tracking; data governance; data storage; brand promotion; patient perception (e.g., safety of devices)
Lack of funding	Obtaining devices; few available for use; ongoing maintenance and resource management; for specific services (e.g., remote services and telehealth)
Macro-level factors	Unclear cost-benefit; unclear what the benefits and relevant populations; unclear what meaningful outcomes and values are in populations of interest

Enablers

Device-related enablers were related to battery, software and interface, wear, costs and privacy. Much of this was the inverse of device-related barriers, and included long battery life, easy to navigate interfaces, easy to analyse data, and cheaper devices. Full summary of device-related enablers in Table 9.

Table 9. Device-related enablers	
Battery	Long battery life
Software and function	Interface easy to navigate and use; easy to analyse and interpret data; easy and quick/real-time data access and download; simple and patient-friendly data outputs; provides regular feedback; personalized data/feedback; multi-purpose devices (i.e., apps and gamification, call functions)
Wear	Small and unobtrusive; attractive
Costs	Cheaper devices; discounts for healthcare-related purchase (i.e., via insurer)
Privacy	Meets confidentiality requirements

Patient-related enablers were related to willingness (of patient), support and accessibility, and knowledge and education. Frequently reported enablers were higher levels of self-efficacy, patients

having their own device, and having a positive relationship with the clinician. Full summary of patient-related enablers in Table 10.

**Table 10. Patient-related enablers**	
Willingness	Higher levels of self-efficacy and engagement with therapy; independent use; views devices as socially acceptable; enjoy and engaged with feedback; perceived safety.
Support and accessibility	Has own device; positive relationship with clinician; support available if needed (e.g., tech issues); access to internet
Knowledge and education	Understand benefits, interest and competence with technology

Clinician and interdisciplinary team-related enablers were related to time factors, skills and training, procedures and systems, greater team involvement, and individual clinician factors. Having time set aside for relevant tasks was considered important, as was training in how to carry out tasks. Availability of defined protocols for use and suitable populations was reported as an enabler, as well as integration of such protocols. Full summary of clinician and interdisciplinary team-related enablers in Table 11.

**Table 11. Clinician and interdisciplinary team-related enablers**	
Time factors	Time allocated for set up; time allocated for analysis
Skills and training	Clinician familiar with use; interpreting/analysing data and outcomes; educating patient; training provided (how to use, benefits of use)
Procedures and systems	Defined protocols for use; defined protocols for suitable patients/populations; integration of protocols and guidelines for use; team members with set responsibilities; relevant information available for clinicians (i.e., for common problems or questions)
Greater team involvement	Used by multiple professions in a multi-disciplinary team; clear leadership support; innovative leadership; technological support; involvement with researchers
Individual clinician factors	Understands value of data; encouraging and supportive clinician; good relationship with patient; use WAMs personally

Healthcare system-related enablers were related to skills and training, procedures and systems, greater team involvement, ethical considerations, funding and resources, and macro-level factors. Enablers relating to skills and training, procedures and systems, and team involvement overlapped with the same clinician and interdisciplinary team-related enablers. More and improved funding was reported as an enabler by many. Many reported enablers existing at a macro-level, with a need for more evidence on relevant outcomes and increased digitisation in healthcare and suitable technological infrastructure. Full summary of healthcare system-related enablers in Table 12.

**Table 12. Healthcare system-related enablers**	
Skills and training	Clinician familiar with use; interpreting/analysing data and outcomes; educating patient; training provided (how to use, benefits of use)
Procedures and systems	Defined protocols for use; defined protocols for suitable patients/populations; integration of protocols and guidelines for use; team members with set responsibilities; relevant information available for clinicians (i.e., for common problems or questions)
Greater team involvement	Used by multiple professions in a multi-disciplinary team; clear leadership support; innovative leadership; technological support; involvement with researchers
Ethical considerations	Aligned with goals of care; devices meet security and privacy requirements
Funding and resources	Improved funding models and quicker approval processes; more devices available; lower device costs
Macro-level	Need for evidence on hospital outcomes (i.e. length of stay, representations); increasing digitisation in healthcare and sophisticated technological infrastructure; advertising and public acceptance; involvement from government

Overall, the response from the first round of surveys was optimistic and revealed interest and enthusiasm for the use of WAMs across a range of clinical populations and settings. Participants considered the use of WAMs in healthcare settings to potentially improve patient care (e.g., improved therapy progression and goal setting), potentially shortened hospital admissions, facilitating home and community-based healthcare, and improved ability to identify service gaps.

It became evident that applications thus far are in their infancy, and require better systems and support for optimal use. Challenges in obtaining funding and the need for more evidence to inform and guide use were highlighted, as was the need for identifying/selecting devices with features suitable for clinical populations.
