# Supplementary material for: Establishing a Consensus-Based Framework for the Use of Wearable Activity Trackers in Health Care: Delphi Study
Source: JMIR Mhealth Uhealth. 2024 Aug 23;12:e55254. doi: 10.2196/55254 (PMC11380062; doi:10.2196/55254)
Supplement: Multimedia Appendix 3 [file mhealth_v12i1e55254_app3.pdf]

Round 2 Results Summary

43 total responses at the time of closing survey = 74.14% response rate

Participants grouped into one of two categories based on professional background: Primary health system (including clinicians, health-system administration); Primarily research. Results displayed for each category, and total sample.

Metrics of the Wearable:

Question 1: For a wearable activity monitor to be useful in clinical settings:

	Healthcare participants					Research participants					All Participants				
	Likert Scale			Total Responses	% of responses = 3	Likert Scale			Total Responses	% of responses = 3	Likert Scale			Total Responses	% of responses = 3
ITEM	1-3	4-6	7-9			1-3	4-6	7-9			1-3	4-6	7-9		
It is critically important for the wearable to measure daily step count	1	6	14	21	**66.67**	0	4	18	22	**81.82**	1	10	32	43	**74.42**
It is critically important for the wearable to measure daily minutes of physical activity	1	1	19	21	**90.48**	0	0	22	22	**100.00**	1	1	41	43	**95.35**
It is critically important for the wearable to measure daily minutes of sedentary time	2	1	18	21	**85.71**	0	5	17	22	**77.27**	2	6	35	43	**81.40**
It is critically important for the wearable to measure daily minutes of sleep	5	10	6	21	**28.57**	2	9	11	22	**50.00**	7	19	17	43	**39.53**
It is critically important for the wearable to measure oxygen saturation	3	13	5	21	**23.81**	11	10	1	22	**4.55**	14	23	6	43	**13.95**
It is critically important for the wearable to measure blood pressure	4	13	4	21	**19.05**	8	12	2	22	**9.09**	12	25	6	43	**13.95**
It is critically important for the wearable to measure heart rate	1	8	12	21	**57.14**	5	10	7	22	**31.82**	6	18	19	43	**44.19**
It is critically important for the wearable to measure the intensity of physical activity (time in different HR zones)	1	7	13	21	**61.90**	4	7	11	22	**50.00**	5	14	24	43	**55.81**
It is critically important for the wearable to measure speed/pace	3	13	5	21	**23.81**	4	12	6	22	**27.27**	7	25	11	43	**25.58**

It is critically important for the wearable to measure energy expenditure	4	11	6	21	**28.57**	7	8	7	22	**31.82**	11	19	13	43	**30.23**
It is critically important for the wearable to measure all daily activities (physical activity, sedentary time and sleep)	2	7	12	21	**57.14**	2	7	13	22	**59.09**	4	14	25	43	**58.14**
It is critically important for the wearable to measure GPS and track location	6	13	2	21	**9.52**	7	14	1	22	**4.55**	13	27	3	43	**6.98**
It is critically important for the wearable to measure body position and posture	5	12	4	21	**19.05**	6	9	7	22	**31.82**	11	21	11	43	**25.58**
It is critically important for the wearable to measure respiratory rate	5	10	6	21	**28.57**	8	14	0	22	**0.00**	13	24	6	43	**13.95**
1-3: not important; 4-6: neutral; 7-9: important															

Question 2: Are there any additional metrics not listed above, which you believe are critically important for a wearable to measure to be useful in clinical settings? *(open response)*

**Different metrics**	Limb position if used to measure range of motion
	Step length
	It would be good to measure UV esp if you want the person to be physically active outdoors and get the benefits of green space and vit D
	On body vs off body (ie. wear) time
	Sunlight exposure may be useful in some clinical settings
	Cadence
	Power. I am a run coach and my clients are often upset if they feel like they have had a slow or ordinary run then I can use their power data to show how hard they worked (as pace, elevation and heart rate might not be accurate)
**More detailed activity data**	Moderate to vigorous PA. Implied within daily PA but this could be light or lightly active with some devices.
	Intensity of physical activity is not mentioned specifically above, although pace/speed is. It would be useful to know the different intensities pf physical activity ie: light, moderate and vigorous physical activity to compare to the physical activity guidelines.
	Would be nice to be able to differentiate between sitting and standing sedentary activity but I don't think any devices can do this
	Number of sit to stand transitions / break from sitting. Maybe (but probably not critically important) the number or duration of prolonged/unbroken sedentary time
**Quality indicators**	Not just totals but indicators of quality and patterns - e.g., pick up long bed rest periods; pick up uneven gaits
	Sleep quality

**Other**	Take raw accelerometer readings and GPS/SPo2/RR/HR ect... you need to create as granular data set as possible. Take raw data and post process...

Question 3: Do you have any further comments on the wearable’s metrics? *(open response)*

**Dependent on patient and purpose**	I've made my decision based on children with chronic disease. HR, BP, RR etc are useful but depends on the clinical population.
	The metrics are largely dependant on the purpose of use - therefore will be different in varied situations
	I think what is critical or not depends on the cohort. I have answered for a chronic pain cohort (focused on activity patterns, reducing sedentary behaviour, increasing PA) This might be very different to a cardiac / respiratory cohort which I presume might be interested in heart rate, O2 etc
	Obviously what different professions view as 'critical' will be different. To me the broad indicators of health outcomes are activity based
**Accuracy**	Questions surrounding accuracy of activity trackers in measuring SpO2, BP and sleep.
	The lack of transparency about how consumer grade devices calculate and smooth activity intensity data reduces the usefulness of this data even when the device appears to calculate minutes of activity well.
	While its nice to consider what would be useful... it will only be really useful if the information is sufficiently accurate/valid, and that is were there might be expected to be difference in clinical populations (or characteristics e.g. slow gait speed) and setting, and characteristics of device wear etc.
	I question the accuracy with todays activity trackers in measuring SpO2, BP, and Sleep.
**Detail of metrics and data**	If wanting to measure sedentary time – need to (i) separate sleep out, and (ii) separate light intensity physical activity (eg. Standing still) from true sedentary behaviour (which occurs in sitting or laying down). Sedentary behaviour is not just ‘inactivity’.
	It is important that certain characteristics of activities should be derivable. For example, the timing of sleep and the bout structure of sedentary time are important. These require epoch-level data. Secondly, day-to-day variability may be important, so multiple days should be captured.
	I don’t believe there is a product that can do all of the above. We are still going to use what allows specific measures. Unless they are shorts / pants that can measure range of motion, physical activity, inactivity, intensity of activity and distance / speeds. A long sleeve t-shirt that tracks UL motion, respiratory motion/rate, cardiac rate, physical activity, inactivity. A cap that does sleep, activity, encephalography
**Additional input of metrics**	Programming with base device; ability to pop up RPE scales if required, during or after activity
**Other**	They need to be linked to public health guidelines and not just marketing gimmicks. They can have both but must be able to link back to the health messages.
	They need to focus on menstrual cycle. I need to be able to explain to a woman that their run might have felt crap because they are in the late luteal phase

Characteristics of the Wearable:

Question 4: Battery and Charging

	Healthcare participants					Research participants					All Participants				
	Likert Scale			Total Responses	% of responses = 3	Likert Scale			Total Responses	% of responses = 3	Likert Scale			Total Responses	% of responses = 3
ITEM	1-3	4-6	7-9			1-3	4-6	7-9			1-3	4-6	7-9		
It is critically important for the wearable to have a battery that lasts at least 2 days on a single charge.	1	4	16	21	76.19	2	4	16	22	72.73	3	8	32	43	74.42
It is critically important for the wearable to have a battery that lasts at least 5 days on a single charge	1	7	13	21	61.90	1	7	14	22	63.64	2	14	27	43	62.79
It is critically important for the wearable to be easy to charge	0	0	21	21	100.00	0	2	20	22	90.91	0	2	41	43	95.35
It is critically important for the wearable to be quick to charge (e.g. reach full charge in 1 hour).	1	6	14	21	66.67	1	5	16	22	72.73	2	11	30	43	69.77
1-3: not important; 4-6: neutral; 7-9: important															

Question 5: How it's worn

	Healthcare participants					Research participants					All Participants				
	Likert Scale			Total Responses	% of responses = 3	Likert Scale			Total Responses	% of responses = 3	Likert Scale			Total Responses	% of responses = 3
ITEM	1-3	4-6	7-9			1-3	4-6	7-9			1-3	4-6	7-9		
It is critically important for the wearable to be comfortable to wear day and night	1	2	18	21	85.71	0	0	22	22	100.00	1	2	40	43	93.02
It is critically important for the wearable to be aesthetically pleasing	1	10	10	21	**47.62**	2	8	12	22	54.55	3	18	22	43	51.16
It is critically important for the wearable to be waterproof	1	4	16	21	76.19	0	3	19	22	86.36	1	7	35	43	81.40
It is critically important for the wearable to have the ability to be	2	6	13	21	61.90	1	13	8	22	**36.36**	3	19	21	43	**48.84**

worn at different bodily sites (other than just the wrist)															
It is critically important for the wearable to be easy to clean and disinfect	1	3	17	21	80.95	0	4	18	22	81.82	1	7	35	43	81.40
1-3: not important; 4-6: neutral; 7-9: important															

Question 6: Data issues

	Healthcare participants					Research participants					All Participants				
	Likert Scale			Total Responses	% of responses = 3	Likert Scale			Total Responses	% of responses = 3	Likert Scale			Total Responses	% of responses = 3
ITEM	1-3	4-6	7-9			1-3	4-6	7-9			1-3	4-6	7-9		
It is critically important for the wearable to have a simple data syncing process	1	0	20	21	95.24	0	0	22	22	100.00	1	0	42	43	97.67
It is critically important for the wearable to have a simple data download/export process	1	0	20	21	95.24	0	3	19	22	86.36	1	3	39	43	90.70
It is critically important that the wearable can measure steps in slow ambulators	1	4	16	21	76.19	1	6	15	22	68.18	2	10	31	43	72.09
It is critically important for the wearable to be able to store at least 5 days of data without syncing/downloading	1	0	20	21	95.24	1	3	18	22	81.82	2	3	38	43	88.37
1-3: not important; 4-6: neutral; 7-9: important															

Question 7: Interface

	Healthcare participants					Research participants					All Participants				
	Likert Scale			Total Responses	% of responses = 3	Likert Scale			Total Responses	% of responses = 3	Likert Scale			Total Responses	% of responses = 3
ITEM	1-3	4-6	7-9			1-3	4-6	7-9			1-3	4-6	7-9		
It is critically important for the wearable to be easy to navigate and set up	0	1	20	21	95.24	1	1	20	22	90.91	1	2	40	43	93.02

It is critically important that personalized goals (e.g. personalized step count) can be set within the wearable (either on the wearable, or within its software).	0	7	14	21	**66.67**	2	7	13	22	**59.09**	2	14	27	43	**62.79**
It is critically important for the wearable to have a simple data analytic interface (e.g. able to interpret key data at a glance)	1	2	18	21	**85.71**	1	7	14	22	**63.64**	2	9	32	43	**74.42**
It is critically important that the wearable can provide instant/real-time feedback to the patient on the wearable (i.e. feedback is available on the wearable itself)	1	5	15	21	**71.43**	0	8	14	22	**63.64**	1	13	29	43	**67.44**
It is critically important that the wearable does NOT provide feedback to the patient on the wearable (i.e. feedback is only available once downloaded to a tablet/computer)	12	8	1	21	**4.76**	12	8	2	22	**9.09**	24	16	3	43	**6.98**
It is critically important for the wearable data to be accessible remotely by the clinician (i.e. that a clinician can monitor activity without physically being with the patient and wearable).	1	8	12	21	**57.14**	0	10	12	22	**54.55**	1	18	24	43	**55.81**
1-3: not important; 4-6: neutral; 7-9: important															

Question 8: Are there any additional device characteristics, not captured above, which you believe are critically important for a wearable to be useful in clinical settings? *(open response)*

**Patient-dependent characteristics and considerations**	The device has to have usability for the user – must perform an essential function in their life – otherwise they won't use it for a prolonged period of time. You have to piggyback onto a device they already have (ie. has a smartphone app).
	I think that comfort is really important and for compliance of older adults
	Interpretations provided by the device need to be patient specific and not generic. They also need to be understandable and easily interpreted by the patient
	Feedback or not for the patient may depend on the own patient and its stage
	My runners run with a pram. Their wrist doesn't move because they are holding the pram. In that instance, they're steps aren't counted! The device must measure steps when holding a pram or mobility device
	That the accuracy of the data is not affected if the person uses a gait aid (i.e. wearables on the wrist / arm are often inaccurate if the patients using a walking frame).

**Purpose-depenent characteristics and considerations**	For studies measuring activity (not an intervention) the option to blind the screen would be good - having a good sized, clear screen is important to encourage behaviour change but if there is an option to turn the screen off it would help in investigations where you're not aiming to change their activity level (reactivity)
	There is no use in targeting these devices for use over a few days. This won't give us the data we need to look at an individuals digital phenotype.
	These questions were a little difficult to answer as I was unsure whether the wearable was to be used by the patient/clinician as a form of feedback, or if the wearable was used as an outcome measure by clinicians.
**Clinically relevant features**	Data to link into current electronic health records systems
	Ability to set reminders for medications or to complete set exercises by the clinician.
	Device tracking (for lost or misplaced devices) or easy to return either in the mail or inbuilt 'return to sender location instructions' if lost.

Question 9: Do you have any further comments on the wearable's characteristics?

**Patient-dependent characteristics and considerations**	Responses based on children, including infants, using wearables. So really important to be able to measure water activity. It would be good if parents could re-charge a wearable.
	If it is complicated on uncomfortable, then it won't be worn and compliance is the only way the patient can benefit from the data.
	Needs to be patient centered - if they wont/cant wear it there is no point
	Again my answers to the second half will depend on the cohort. These devices should be used with great care in people with eating disorders, disordered eating and/or shape and weight concerns. If they are to be used at all (and this would need great consideration) the feedback should not be immediately available to the participant.
**Purpose-dependent characteristics and considerations**	Some characteristics vary according to purpose – to motivate I want participants to see output, for baseline assessment I don't want them to see output
	Would only need participant to be blind to data if used as measurement tool - e.g. evaluation of a programme - rather than clinically. Could use other tools for this, thus allowing the WAM to be focused on the key things of benefit for clinical use
	The importance of whether feedback is available to the patient depends on the purpose of using the WAM i.e. if it's to increase PA (interventional) then yes - feedback is needed. If it's to evaluate something (observational), then feedback to the patient is not needed).
	The characteristics will really depend on the way the wearable is being used. If it is being used as a motivational instrument, obviously wearer-accessible feedback is important. If it is being used a a monitoring tool, that's not so important.
	The option to choose or switch between real-time feedback to patients and hidden (blinded) results in a single device would be good - thus could suit 'intervention' delivery/testing (where device informed goal setting etc is available), but also more research purposes where you might want participant blinded
**Other**	Cost as low as possible; interface capabilities with a range of devices; clinician owned or patient owned. Alert/alarm functionality.
	I hesitated for battery time. A minimum of 3 days would mean it would last the weekend safely.
	Ideal if the person could add 'time stamps' to indicate sleep etc...getting rid of sleep time is often a problem.
	Further, if it is hard to interpret, then they will not collect data if no one is using the data or feeding back / intervening based on the collected data

Question 10: Taking all things into account (accuracy, convenience, hygiene etc) which bodily wear site is most appropriate?

	Healthcare participants		Research participants		All Participants	
**Response**	**Number**	**Percent**	**Number**	**Percent**	**Number**	**Percent**
Wrist	15	**71.43**	16	**72.73**	31	**72.09**
Hip	2	**9.52**	2	**9.09**	4	**9.30**
Thigh	1	**4.76**	3	**13.64**	4	**9.30**
Shoe	0	**0.00**	0	**0.00**	0	**0.00**
Ankle	1	**4.76**	1	**4.55**	2	**4.65**
Trunk	2	**9.52**	0	**0.00**	2	**4.65**

Question 11: Do you have any further comments on the wearable's bodily wear site?

**Patient-dependent wear site**	For infants hip/pelvis is good as you get whole body measure. Ankle is ok but only tells you about leg movement.
	Wrist is likely most acceptable to patients and carers re utilization of device and ease of application
	Hip/thigh/trunk most accurate but not comfortable/feasible. The convenience of wrist worn should outweigh any inaccuracies, as it is better for the patient to actually wear than be left on the side.
	I would choose wear site based on patient age/reliability, young children and some adolescents are so likely to lose the wrist worn device, which inhibits benefits of using due to cost.
	Depends on the patient – shoe might be more appropriate if the participants are reliant on walking aids.
	Sometimes if the patient is on a special event may he or she would like to be able to change the wrist by the ankle or another non visible site
	Certain populations can't wear on wrist, eg. healthcare workers, so might be good to have an alternate option.
	It really depends on the population we are wanting to study
	Not pocket as women's clothes seldom have pockets
	Patients in hospitals don't often have/wear shoes - so not likely to be able to fit… I like wrist, but, patients in hospital tend to have lots of attachments/lines/dressings in/around their wrist (and fragile skin) so that can be a practically difficult location (esp if needs to be on non-dominant hand... might not be an option). Thus I think thigh on balance is good (especially for sedentary behaviour); I have no experience with ankle, but that might also be appropriate (especially for steps)
	Again it depends on the cohort and what you want to measure. If you want to distinguish sitting and lying from standing then you need to wear it on the thigh. However if you are more interested in activity levels and heart rate then the wrist might be more convenient.
	For people who don't want to wear it during sleep, it should act as a nearable where heart rate is still monitored by the device while it sits on the bedside table
**Purpose-dependent wear site**	If the wearable is used as an outcome measure, the high or thigh would be best. If it is to be used for immediate feedback to the patient, wrist would be best.
	Centre of gravity for location related to movement, as this saves on relying upon the arm or leg swing to generate clicks. Thorax if you are looking for physiological changes to cardio respiratory system

	Need to be able to separate standing still (which is light intensity PA) from sedentary time – and the only way to do this is with a device that includes a thigh inclinometer
**Other comments**	Hip/waist or ankle are almost as good as wrist
	It doesn’t matter
	Wrist is convenient and conspicuous

Patient Populations

Question 12: Patient Populations

ITEM	Healthcare participants – Likert Scale 1-3	4-6	7-9	Total Responses	% of responses = 3	Research participants – Likert Scale 1-3	4-6	7-9	Total Responses	% of responses = 3	All Participants – Likert Scale 1-3	4-6	7-9	Total Responses	% of responses = 3
Wearables are highly appropriate for use with orthopaedic cohorts	2	1	18	21	**85.71**	1	7	14	22	**63.64**	3	8	32	43	**74.42**
Wearables are highly appropriate for use with neurological cohorts	0	2	19	21	**90.48**	0	9	13	22	**59.09**	0	11	32	43	**74.42**
Wearables are highly appropriate for use with cardiovascular cohorts	0	0	21	21	**100.00**	0	2	20	22	**90.91**	0	2	41	43	**95.35**
Wearables are highly appropriate for use with pulmonary cohorts	0	0	21	21	**100.00**	0	5	17	22	**77.27**	0	5	38	43	**88.37**
Wearables are highly appropriate for use with metabolic cohorts (e.g. obesity, bariatrics, diabetes)	0	0	21	21	**100.00**	0	1	21	22	**95.45**	0	1	42	43	**97.67**
Wearables are highly appropriate for use with mixed rehabilitation cohorts	0	2	19	21	**90.48**	0	4	18	22	**81.82**	0	6	37	43	**86.05**
Wearables are highly appropriate for use with ICU and critical care cohorts	6	9	6	21	**28.57**	7	9	6	22	**27.27**	13	18	12	43	**27.91**
Wearables are highly appropriate for use with chronic pain cohorts	0	2	19	21	**90.48**	2	5	15	22	**68.18**	2	7	34	43	**79.07**
Wearables are highly appropriate for use with older adult cohorts (geriatrics and aged care)	0	1	20	21	**95.24**	1	3	18	22	**81.82**	1	4	38	43	**88.37**
Wearables are highly appropriate for use with paediatric cohorts	0	6	15	21	**71.43**	4	5	13	22	**59.09**	4	11	28	43	**65.12**
1-3: not important; 4-6: neutral; 7-9: important															

Question 13: Are there any further hospital-related patient populations for which wearables are highly appropriate?

**Additional cohorts**	Trauma. Especially those who had an accident during physical activity
	Mental Health
	Mental health patients

	Recovery from cancer.
	Oncology, burns - although they could be considered "mixed rehab". Ideally, PA is important for everyone, across the lifespan!
	Does the definition for cardiovascular and surgical cohorts include cardiac and pulmonary surgery?... What about other general surgical (e.g. abdo surg) cohorts, or oncology services?
	All patients/their relatives and all staff. Use the wearable devices to track nurses to improve their efficiency (nursing time is the biggest cost in healthcare)
**Comments on setting**	Hospital in the home/ community patients
	Outpatients and hospital in the home patients too
**Other comments**	Note ICU/ crit care monitored anyway, does a wearable add great value in these highly monitored units, unless it is to replace current monitoring
	I am not a hospital clinician, so above is just my guesses as how a PA wearable could be used clinically

Question 14: Do you have any further comments on the suitability of wearables in specific patient populations?

**Suitability for any and varied populations**	Believe they could be appropriate for any cohort (have seen them used in all the above), but consideration would have to be taken in wear instructions, wear-site, and interpretation of output
	Appropriate for any population where you wish to track something objective relevant to the rehabilitation
	People who are just interested in improving their health (i.e.They want to start running but have congenital abnormality or arthritis etc)
**Specific population and patient considerations**	I only rated ICU lower for practical reasons (needing to take them off for imaging, patients ability to care for them etc.)
	Please see previous comment regarding participants with eating concerns: Again my answers to the second half will depend on the cohort. These devices should be used with great care in people with eating disorders, disordered eating and/or shape and weight concerns. If they are to be used at all (and this would need great consideration) the feedback should not be immediately available to the participant.)
	Our use with older adults needs to be expanded and standardized
**Other**	For some of these - if issues around sleep monitoring accuracy with actigraphy was better it might be of more interest and use (e.g. ICU/critical care)

Healthcare Settings

Question 15: Measuring Activity (ie. physical activity +/- sedentary behaviour +/- sleep)

ITEM	Healthcare participants – Likert Scale 1-3	4-6	7-9	Total Responses	% of responses = 3	Research participants – Likert Scale 1-3	4-6	7-9	Total Responses	% of responses = 3	All Participants – Likert Scale 1-3	4-6	7-9	Total Responses	% of responses = 3
Measuring activity prior to an elective hospital admission	1	8	12	21	57.14	1	7	14	22	63.64	2	15	26	43	60.47
Measuring activity during an inpatient hospital admission	0	5	16	21	76.19	3	5	14	22	63.64	3	10	30	43	69.77
Measuring activity during home-based rehabilitation	0	0	21	21	100.00	0	2	20	22	90.91	0	2	41	43	95.35
Measuring activity following a hospital admission (i.e. after discharge from hospital)	0	3	18	21	85.71	0	4	18	22	81.82	0	7	36	43	83.72
Measuring activity in an outpatient setting	0	1	20	21	95.24	0	4	18	22	81.82	0	5	38	43	88.37
Measuring activity in a community-based setting (inside or outside the home)	0	1	20	21	95.24	0	1	21	22	95.45	0	2	41	43	95.35
Measuring activity in residential aged-care setting (including home-based care, or live-in care facilities)	0	4	17	21	80.95	0	4	18	22	81.82	0	8	35	43	81.40

1-3: not important; 4-6: neutral; 7-9: important

Question 16: Measuring physiological parameters (eg. Heart rate, respiratory rate, oxygen saturation)

ITEM	Healthcare participants – Likert Scale 1-3	4-6	7-9	Total Responses	% of responses = 3	Research participants – Likert Scale 1-3	4-6	7-9	Total Responses	% of responses = 3	All Participants – Likert Scale 1-3	4-6	7-9	Total Responses	% of responses = 3
Measuring physiological parameters prior to a known (elective) hospital admission	0	10	11	21	52.38	3	10	9	22	40.91	3	20	20	43	46.51
Measuring physiological parameters during an inpatient hospital admission	0	9	12	21	57.14	2	8	12	22	54.55	2	17	24	43	55.81

	Healthcare participants					Research participants					All Participants				
Measuring physiological parameters during home-based rehabilitation	0	2	19	21	90.48	0	8	14	22	63.64	0	10	33	43	76.74
Measuring physiological parameters following a hospital admission	0	5	16	21	76.19	0	8	14	22	63.64	0	13	30	43	69.77
Measuring physiological parameters in an outpatient setting	1	4	16	21	76.19	1	9	12	22	54.55	2	13	28	43	65.12
Measuring physiological parameters in a community-based setting (inside or outside the home)	0	4	17	21	80.95	1	7	14	22	63.64	1	11	31	43	72.09
Measuring physiological parameters in residential aged-care setting (including home-based care, or live-in care facilities)	0	7	14	21	66.67	3	5	14	22	63.64	3	12	28	43	65.12
1-3: not important; 4-6: neutral; 7-9: important															

Question 17: Intervening on Activity

	Healthcare participants					Research participants					All Participants				
	Likert Scale			Total Responses	% of responses = 3	Likert Scale			Total Responses	% of responses = 3	Likert Scale			Total Responses	% of responses = 3
**ITEM**	1-3	4-6	7-9			1-3	4-6	7-9			1-3	4-6	7-9		
Intervening on activity prior to an elective hospital admission	0	8	13	21	61.90	4	4	14	22	63.64	4	12	27	43	62.79
Intervening on activity during an inpatient hospital admission	1	7	13	21	61.90	4	6	12	22	54.55	5	13	25	43	58.14
Intervening on activity during home rehab (inpatient classification)	0	3	18	21	85.71	0	3	19	22	86.36	0	6	37	43	86.05
Intervening on activity following a hospital admission	0	5	16	21	76.19	2	5	15	22	68.18	2	10	31	43	72.09
Intervening on activity in an outpatient setting	0	5	16	21	76.19	0	5	17	22	77.27	0	10	33	43	76.74
Intervening on activity in a community-based setting (inside or outside the home)	0	4	17	21	80.95	0	2	20	22	90.91	0	6	37	43	86.05
Intervening on activity in residential aged-care setting (including home-based care, or live-in care facilities)	0	7	14	21	66.67	1	4	17	22	77.27	1	11	31	43	72.09
1-3: not important; 4-6: neutral; 7-9: important															

Question 18: Are there any further healthcare settings for which wearables are highly appropriate? Is this for measurement, or for intervention?

**Additional settings**	I don't only work in healthcare settings, but in disability - really important for intervention
	Gym settings where physios work with personal trainers
**Suitable for any setting**	I would not differentiate between settings, if it is useful in one it is likey useful in
**Accuracy limitations**	I don't really see the 'value' in the physiological monitoring, unless..... it is sufficiently accurate as compared to usual devices in the hospital setting (or could reduce the need to patients to be attached to so many things with a single device), it could provide that accurate continuous physiological monitoring for exercise safety in acutely unwell/fragile populations, or (in the post-discharge sense). In the post D/C phase, would be most useful if there was some sort of 'flag' or way that patients who are unwell (e.g. consistently abnormal obs) or who don't demonstrate sufficient 'recovery' of their activity on discharge could be flagged to health professionals as at risk and potentially hospital re-admission avoidance strategies could be targeted/implemented.
**Evidence limitations**	They should be worn pre=during and post hospital stay… however we don’t have enough evidence on their ability to alter behaviour

Use of wearable activity monitors in clinical settings to date

Question 19: Do you currently, or have you previously used wearable activity monitors in clinical settings?

	Healthcare participants		Research participants		All Participants	
**Response**	**Number**	**Percent**	**Number**	**Percent**	**Number**	**Percent**
Yes - currently using	11	**52.38**	4	**18.18**	15	**34.88**
Yes - previously used	5	**23.81**	7	**31.82**	12	**27.91**
No, I don't have direct experience of using wearable activity monitors in clinical settings	5	**23.81**	11	**50.00**	16	**37.21**

Question 20: Which wearable device or devices have you used in CLINICAL SETTINGS? (select all that apply)

	Healthcare participants	Research participants	All Participants
**Response**	**Number**	**Number**	**Number**
Fitbit Zip	2	2	4
Fitbit Charge	3	4	7
Other Fitbit	7	1	8
Apple Watch	4	2	6
Garmin Vivoactive	1	0	1
Garmin Vivofit	2	0	2
Polar Watch	4	1	5
Basic Pedometer	5	2	7
Smartphone Pedometer App	4	1	5
Actical Accelerometer	0	0	0
GeneActivAccelerometer	0	5	5
ActigGraph Accelerometer	2	4	6
Axivity Accelerometer	0	0	0
Other (please specify)	6	5	0

	ActivPAL (3)<br>GPS tracker<br>StepWatch<br>Other Fitbit	ActivPAL (2)<br>Step Activity Monitor<br>Positional Activity Logger ver. 1<br>Garmin Fenix	

Question 21: Please tell us about the software you have used to download and process the data. Is it proprietary software linked directly to the wearable (eg. the Fitbit app), or do you use some kind of third party software (and if so, what is the name of the software)? Can you view multiple patients' data easily, or do you have to log into separate accounts to view different patients' data? Please provide as much detail as possible.

**Response**	**Devices Used**
ActivPal allows accurate measurement of SB and position changes Need to log into separate accounts	ActivPAL
Proprietary software for both Geneactive and ActivPAL, plus, previously (at the time) used some custom software from UniSA via *** (?some sort of custom Matlab program?). I can't recall much about the data viewing, but think it was only able to look at a single patient file at a time. Whatever it was... almost nothing was easy (an no opportunity to customise analysis/view, e.g. unable to set different analysis periods or analysis cut-points)	ActivPAL, GeneActiv Accelerometer
Fitbit app	Other Fitbit
Fitabase, manufacturing proprietary software	N/A in clinical settings
Software linked to the app	Other Fitbit
Both - expensive devices such as the SAM have high quality software programs. Cheaper devices like the Fitbit do not, and we have used the expertise of colleagues to write extraction software in Matlab	Fitbit Zip, Fitbit Charge, Other Fitbit, Polar Watch, GeneActiv Accelerometer, Actigraph Accelerometer, Step Activity Monitor
Polar	Polar Watch, Smartphone Pedometer App
We generally use in-house custom software, Cobra	GeneActiv, Actiggraph Accelerometer
Actilife software. Just used on wrist to measure how long spent exercising	Actigraph Accelerometer
Linked to the patient's smart phone. Not to my own hardware. Patient references their own content and records for self	Fitbit Charge, Apple Watch, Garmin Vivofit, Polar Watch, Basic Pedometer, Smartphone Pedometer App
Garmin	Garmin Vivofit

Fitbit app - need to log into each patient's account separately - this was time consuming and more difficult	Fitbit Charge
The Positional Activity Logger Version 1 preceded current activity monitors and measured uptime. It used custom software for downloading uptime over a 24 hour period.	Positional Activity Logger ver 1
Internally developed software but it was not very user friendly.	Fitbit Charge, GeneActiv Accelerometer
For clinical purposes, I use a pedometer for walking based targets as part of a home exercise program	Basic Pedometer
ActivPAL has its own software that is downloaded to a computer. Software can't be downloaded on multiple computers. Clinician needs to be able to access the computer that has the software. Is time consuming having to move to THE computer from a ward area and then back to the patient. Graphics that display the output is really user friendly (colour coded) and easy for the child/family to understand.	ActivPAL
Fitbit software on ipad, polar watch used as immediate feedback, at time utilized the software to PC and download was clunky	Other Fitbit, Polar Watch, Basic Pedometer,
Propitiatory Software for both Fitbit (one and flex) and GPS tracker	Other Fitbit, Smartphone Pedometer App, GPS tracker
GeneActiv software is provided and useful to download data. Cobra coding software was useful to analyse different activity intensity	GeneActiv Accelerometer
ActiLife to process the ActiGraph data, which is reasonably easy to use. I have not accessed the Fitbit data	Fitbit Chards, ActiGraph Accelerometer
Proprietary software, own app to export data from Fitbit API	Fitbit Zip, Apple Watch, Basic Pedometer, Smartphone Pedometer App, Actigraph Accelerometer
I provide the actigraph monitor and download data from their software - I don't find it particularly user friendly, and I find it difficult to get data in a useful format for feedback to the patient. The other devices I will use are the one patients already own - I check they have their device set up (proper age/weight/height etc) and help them set their daily goals and make sure they know how to get to their own data on their own phone app. I then get them to feed that data back to me during appointments - my focus is on having the patient have control and understanding of how to use the device to help them implement lifestyle change recommended. Fitbit and Apple are the only two I have a reasonable understanding of setup, if patients have other devices I direct them back to their own device setup to DIY. I don't look at patient data as a group, it is always looked at individually and apart from Actigraph, it is always with the patient present.	Other Fitbit, Apple Watch, Smartphone Pedometer App, ActiGraph Accelerometer
Not used in a clinical setting (previous question about devices used applies to workplace settings only) Fitabase and the fitbit app was used in all my projects. With Fitabase you can view and download each individual's data from one place, participant just needs to agree to share data with fitabase and you can get every metric fitbits collect at 1sec, 60sec, hour and daily intervals. Pretty expensive to use (we paid $2500) but worked very well	Fitbit Charge
I only have experience with polar watch for hr and Fitbit app monitoring single patients during step tests or momentary measures	Fitbit Charge, Other Fitbit, Apple Watch, Polar Watch, Basic Pedometer,
The activPAL is a small triaxial accelerometer that you can affix to thigh with waterproof dressings. It measures up to 10 days data (sedentary, PA and sleep). It has its own associated software. Once you download the data from the device you can view it in a user-friendly PDF format or detailed data as a CSV file (for research). You can view any activPAL data from the one software program/login.	ActivPAL
Inhouse - data fed to Matlab analytics/display	N/A in clinical settings

Software directly linked to wearable. Usually had to log in to separate accounts. Could see all data together including time/usage etc - but needed to pay FitBit to get download of metrics. With StepWatch need a particular programme - do not see the data in real time, no immediate feedback for pt, mainly used in research	Fitbit Zip, Other Fitbit, Basic Pedometer, StepWatch
Patient takes screenshot of their data and sends to me. Otherwise I monitor via strava as their device syncs to it	Apple Watch, Garmin Fenix
Actilife	ActiGraph Accelerometer

Software

Question 22: How satisfied are you with the software for downloading/processing/interpreting patient's data?

**Likert Scale**	**1**	**2**	**3**	**4**	**5**	**6**	**7**	**8**	**9**	**Total number of responses**
Responses	2	0	1	3	11	2	5	6	2	32
Percentage	6.25%	0%	3.13%	9.38%	34.38%	6.25%	15.63%	18.75%	6.25%	

Question 23: If you answered 8 or below, how could the software be different/improved to better meet the needs of clinicians and patients in clinical use?

**Wireless download**	Wireless download
	It's via USB port only not wifi/Bluetooth
**Capacity for changing interface and data display, and input of self-report data**	Visual displays at various levels of simplicity
	Presented in a meaningful format, easy to present to pt
	Be explained in laypersons terms and be able to choose exactly the parameters we want to share and graph. Need to be able to provide feedback too
	Limited data views. Ability to select different parameters of interest to view
	Interactive interfaces with switch on/off compatibility of base station tools would be useful e.g. pop ups of VAS for pain and fatigue, in response to exercise. Also please see previous comment re RPE measures. Automatic physiological measurement in response to activity thresholds could also be useful in some circumstances. e.g. cardiac rehab
**Centralised access to multiple patient data**	Having all participant data accessible in one central location/login
	Ability to track different patients at different time
**Improved export/download processes**	Downloading data needs to be easy, instant and in a format that patients will understand and respond to
	The software we used could only download one patient at a time, it would be better if could do batch download.
**Access to raw data sets/more detailed data,**	Need external analysis to determine length of SB bouts
	Editable flexible
	I just need raw data. Python does the rest

**and ability to conduct separate analyses**	Being able to analyse and manipulate the data in software would make the process easier and also reduce the risk of errors by transferring data
	Custom for specific interventions
	I went in the middle because the range of software is huge. For things like the SAM I would answer 8 or 9. for fitbit zip etc data is not detailed enough to interpret a lot of the information that we want to extract
	Ideally I would like to receive the data from the patients' own device but have it presented in a table format which allowed for multiple patients data on one page. I don't know that the hospital data privacy/security would allow that as the data comes via a 3rd party. For my current clinical needs I think the process I use at present is adequate.
**Other**	We had some issues syncing our data together with timestamps, I think it was more of an issue for our inability to use excel and access but we struggled to get all the data from each participant matched. Daylight savings happened during our project which didn't help...
	Could give an idea of steps across the day
	Always a worry if a device malfunction, if knew it was recording or not until got the data.
	Uptake of HR real-time data can be a bit slow with wrist monitors
