# Supplementary material for: Establishing a Consensus-Based Framework for the Use of Wearable Activity Trackers in Health Care: Delphi Study
Source: JMIR Mhealth Uhealth. 2024 Aug 23;12:e55254. doi: 10.2196/55254 (PMC11380062; doi:10.2196/55254)
Supplement: Multimedia Appendix 4 [file mhealth_v12i1e55254_app4.pdf]

Round 3 Results

43 total responses at the time of closing survey = 74.14% response rate
Participants grouped into one of two categories based on professional background: Primary health system (including clinicians, health-system administration); Primarily research. Results displayed for each category, and total sample.

Section 1: Re-rating items where consensus was not reached, and rating new suggested items
Metrics
Question 1: For a wearable to be useful in clinical settings (re-rated)

ITEM	Healthcare participants					Research participants					All Participants				
	Likert Scale			Total Responses	% of responses = 3	Likert Scale			Total Responses	% of responses = 3	Likert Scale			Total Responses	% of responses = 3
	1-3	4-6	7-9			1-3	4-6	7-9			1-3	4-6	7-9		
It is critically important for the wearable to measure daily step count	1	2	16	19	84.21	0	3	21	24	87.50	1	5	37	43	86.05
It is critically important for the wearable to measure daily minutes of sleep	2	10	7	19	**36.84**	3	13	8	24	**33.33**	5	23	15	43	**34.88**
It is critically important for the wearable to measure heart rate	0	2	17	19	89.47	6	10	8	24	**33.33**	6	12	25	43	58.14

Question 2: For a wearable to be useful in clinical settings (new item)

	Healthcare participants					Research participants					All Participants				
	Likert Scale			Total Responses	% of responses = 3	Likert Scale			Total Responses	% of responses = 3	Likert Scale			Total Responses	% of responses = 3
ITEM	1-3	4-6	7-9			1-3	4-6	7-9			1-3	4-6	7-9		
It is critically important for the wearable to measure limb position (e.g. to measure range of motion)	6	12	1	19	**5.26**	14	8	2	24	**8.33**	20	20	3	43	**6.98**
It is critically important for the wearable to measure step length	5	12	2	19	**10.53**	11	8	5	24	**20.83**	16	20	7	43	**16.28**
It is critically important for the wearable to measure ultraviolet/sunlight exposure	10	7	2	19	**10.53**	14	9	1	24	**4.17**	24	16	3	43	**6.98**
It is critically important for the wearable to measure on vs. off body time (e.g. wear time)	2	10	7	19	**36.84**	0	5	19	24	**79.17**	2	15	26	43	**60.47**
It is critically important for the wearable to measure cadence	4	12	3	19	**15.79**	5	10	9	24	**37.50**	9	22	12	43	**27.91**
It is critically important for the wearable to measure power	7	11	1	19	**5.26**	11	11	2	24	**8.33**	18	22	3	43	**6.98**
It is critically important for the wearable to measure sit to stand transitions	1	7	11	19	**57.89**	1	9	14	24	**58.33**	2	16	25	43	**58.14**
It is critically important for the wearable to capture the position of sedentary behaviour (e.g. sitting/standing/lying down)	1	8	10	19	**52.63**	1	9	14	24	**58.33**	2	17	24	43	**55.81**
It is critically important for the wearable to specify details of physical activity intensities (e.g. light/moderate/vigorous)	0	4	15	19	**78.95**	1	6	17	24	**70.83**	1	10	32	43	**74.42**
It is critically important for the wearable to factor in the menstrual cycle	8	7	4	19	**21.05**	11	10	3	24	**12.50**	19	17	7	43	**16.28**

Characteristics of the wearable

Question 3: For a wearable to be useful in clinical settings (re-rated)

	Healthcare participants					Research participants					All Participants				
	Likert Scale			Total Responses	% of responses = 3	Likert Scale			Total Responses	% of responses = 3	Likert Scale			Total Responses	% of responses = 3
ITEM	1-3	4-6	7-9			1-3	4-6	7-9			1-3	4-6	7-9		
It is critically important for the wearable to be aesthetically pleasing	1	7	8	16	50	3	9	12	24	50	4	16	20	40	50

Question 4: For a wearable to be useful in clinical settings (new item)

	Healthcare participants					Research participants					All Participants				
	Likert Scale			Total Responses	% of responses = 3	Likert Scale			Total Responses	% of responses = 3	Likert Scale			Total Responses	% of responses = 3
ITEM	1-3	4-6	7-9			1-3	4-6	7-9			1-3	4-6	7-9		
It is critically important for the wearable to have an option to 'hide' the interface from the patient	4	7	8	19	**42.11**	3	7	14	24	58.33	7	14	22	43	51.16
It is critically important for the wearable data to link with electronic health records	4	6	9	19	**47.37**	4	6	14	24	58.33	8	12	23	43	53.49
It is critically important for the wearable to have the ability to set specific reminders (e.g. for medications, to compete prescribed exercises)	1	7	11	19	57.89	2	9	13	24	54.17	3	16	24	43	55.81
It is critically important for the wearable to have device tracking capabilities (e.g. for lost devices)	0	6	13	19	68.42	2	14	8	24	**33.33**	2	20	21	43	**48.84**
It is critically important for the wearable to provide data that is easy to interpret	0	3	16	19	84.21	2	0	22	24	91.67	2	3	38	43	88.37
It is critically important for the wearable to have the capacity to select between different metrics for viewing	0	3	16	19	84.21	3	7	14	24	58.33	3	10	30	43	69.77

Wear site

Question 5: For a wearable to be useful in clinical settings (re-rated)

ITEM	Healthcare participants					Research participants					All Participants				
	Likert Scale			Total Responses	% of responses = 3	Likert Scale			Total Responses	% of responses = 3	Likert Scale			Total Responses	% of responses = 3
	1-3	4-6	7-9			1-3	4-6	7-9			1-3	4-6	7-9		
It is critically important for the wearable to have the ability to be worn at different bodily sites (other than just the wrist)	0	5	14	19	73.68	3	8	13	24	54.17	3	13	27	43	62.79

Question 6: For a wearable to be useful in clinical settings (new item)

ITEM	Healthcare participants					Research participants					All Participants				
	Likert Scale			Total Responses	% of responses = 3	Likert Scale			Total Responses	% of responses = 3	Likert Scale			Total Responses	% of responses = 3
	1-3	4-6	7-9			1-3	4-6	7-9			1-3	4-6	7-9		
Wrist is likely the most appropriate wear site for patient acceptability and compliance	0	4	15	19	78.95	2	4	18	24	75.00	2	8	33	43	76.74
Wear site may need to be adapted in some populations and individuals (e.g. those with walking frames)	0	2	17	19	89.47	1	4	19	24	79.17	1	6	36	43	83.72
Wear site may need to be adapted for different purposes (e.g. assessment vs. intervention)	0	6	13	19	68.42	1	12	11	24	45.83	1	18	24	43	55.81

Suitable patient populations
Question 7: Patient populations (new item)

ITEM	Healthcare participants					Research participants					All Participants				
	Likert Scale			Total Responses	% of responses = 3	Likert Scale			Total Responses	% of responses = 3	Likert Scale			Total Responses	% of responses = 3
	1-3	4-6	7-9			1-3	4-6	7-9			1-3	4-6	7-9		
Wearables are highly appropriate for use with oncology cohorts	0	4	15	19	78.95	0	9	15	24	62.50	0	13	30	43	69.77
Wearables are highly appropriate for use with mental health cohorts	0	7	12	19	63.16	1	7	16	24	66.67	1	14	28	43	65.12
Wearables are highly appropriate for use within the disability sector	0	4	15	19	78.95	0	8	16	24	66.67	0	12	31	43	72.09

Suitable settings and use cases
Question 8: Wearables are highly appropriate for (re-rated)

ITEM	Healthcare participants					Research participants					All Participants				
	Likert Scale			Total Responses	% of responses = 3	Likert Scale			Total Responses	% of responses = 3	Likert Scale			Total Responses	% of responses = 3
	1-3	4-6	7-9			1-3	4-6	7-9			1-3	4-6	7-9		
Measuring physiological parameters prior to a known (elective) hospital admission	0	6	13	19	68.42	2	7	15	24	62.50	2	13	28	43	65.12

Section 2: Software

Question 9: For software to be 'ideal' for supporting wearables in clinical settings

	Healthcare participants					Research participants					All Participants				
	Likert Scale			Total Responses	% of responses = 3	Likert Scale			Total Responses	% of responses = 3	Likert Scale			Total Responses	% of responses = 3
ITEM	1-3	4-6	7-9			1-3	4-6	7-9			1-3	4-6	7-9		
It is critically important for the wearable to have the capacity to input self-report data (e.g. RPE, pain and fatigue VAS)	0	8	11	19	57.89	2	10	12	24	50.00	2	18	23	43	53.49
It is critically important for the wearable to provide useful and relevant data on the wearable interface (e.g. data provided on device screen and/or associated application can be used as is, without further analysis needed)	0	6	13	19	68.42	0	7	17	24	70.83	0	13	30	43	69.77
It is critically important for the wearable to have the capacity to present relevant data at varying levels of simplicity (e.g. simple display for patient, and more comprehensive display for clinician)	0	1	18	19	94.74	0	2	22	24	91.67	0	3	40	43	93.02
It is critically important for the software to enable centralized access of patient data (e.g. view patient data remotely from hospital/clinic)	0	5	14	19	73.68	0	2	22	24	91.67	0	7	36	43	83.72
It is critically important for the software to enable access to multiple patients' data simultaneously (e.g. clinician having the ability to switch the patient data they are viewing without having to log in/out of different accounts)	0	5	14	19	73.68	0	4	20	24	83.33	0	9	34	43	79.07
It is critically important for the software to enable wireless data upload	0	1	18	19	94.74	0	2	22	24	91.67	0	3	40	43	93.02
It is critically important for the software to enable batch data downloads (e.g. multiple patients at a time)	0	3	16	19	84.21	0	4	20	24	83.33	0	7	36	43	83.72

It is critically important for the software to enable the clinician to access raw data sets obtained by the wearable (e.g. for analysis, to add timestamps, to identify non-wear time)	0	4	15	19	78.95	2	4	18	24	75.00	2	8	33	43	76.74
It is critically important for the software to enable the clinician to conduct separate/additional analyses to those provided by the wearable	0	4	15	19	78.95	1	4	19	24	79.17	1	8	34	43	79.07
It is critically important for the software to have the capacity to download and access data sets instantly	0	6	13	19	68.42	0	6	18	24	75.00	0	12	31	43	72.09
It is critically important for the software to have the capacity to automatically present data in different formats (e.g. provides graphs, tables, scores without clinician having to edit and analyse data sets)	0	9	10	19	52.63	0	6	18	24	75.00	0	15	28	43	65.12

Question 10: (optional) Do you have any further comments on software?

	Related to the software, questions in part 1 of this survey were about sleep duration via the tracker. this is easily quantifiable but healthy sleep is more than that. so important for people to be able to have the subjective aspects entered into it - eg perceived quality. this falls into above question about user entered metrics. my comments on tracking steps based on fact it is a easily quantifiable metric that almost all people "get" though some issues with some disability populations
	Systems need to be readability friendly not technologically oriented as a front end
	These are difficult to answer. It really depends on how you plan to use the wearable
	Wearer compliance requires the wearable to be easily interpreted by the patient and data uploads are simple to perform by the patient
	By “instantly” do you mean in real time or just that the downloading is fast (which is what I assume this means)? It’s more helpful to have data from the device being uploaded/synced constantly rather than uploaded in infrequent batches (i.e. at night when it’s plugged in) as then you can know quickly if someone isn’t wearing the device or something has gone wrong with the syncing.
	Adherence to standards for data and interoperability; attention to error/failure mode detection and warnings
	It must be free at least for the patients

Section 3: Barriers and strategies to address them

Patient-related barriers

Question 11: What do you think is the most significant category of patient-related barrier?

Patient-related barrier	Healthcare participants		Research participants		All Participants	
	Total Responses	% Responses	Total Responses	% Responses	Total Responses	% Responses
Clinical unsuitability	7	36.84	5	20.83	12	27.91
Patient reluctance	4	21.05	9	37.50	13	30.23
Resource barriers	5	26.32	6	25.00	11	25.58
Poor health and/or digital literacy	3	15.79	4	16.67	7	16.28

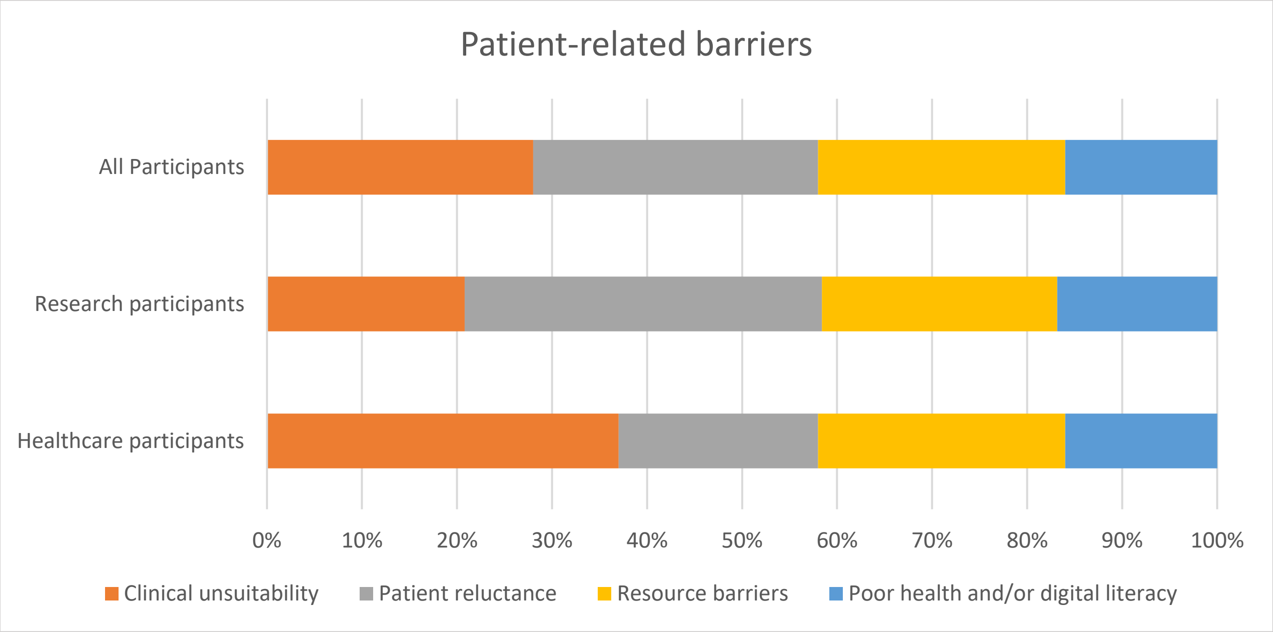

Question 12: (optional) What are some strategies to address this category of barrier?

**Clinical unsuitability**	Have a structured approach to prescription of the device (e.g. clinician needs to be aware of the recovery stage of a person with an eating disorder and if the device might trigger behaviours not conducive with recovery [i.e. calorie counting])
	Potentially having different device for specific needs
**Patient reluctance**	Compliance strategies; patient education
	Address underlying barriers for pt in engaging with tech, probably same as barriers to engaging in exercise - identify goals, discrepancies, strategies to assist in engagement
	Pay them
	Education/information
	MI to understand concerns.
	Ensuring patients understand the use of the data, that its use is to only enhance rehab, not analyse lifestyle choices. reassure re: privacy and use a device number not patient name, for example.
	This is like stages of change. If they aren't interested, they won't do it. Focus on those with some interest at least and educate, etc.
**Resource barriers**	Health fund to cover cost
	Grant funding: recognition by health services that measurement of PA is part of intervention where there is evidence that PA is related to pt outcomes, e.g. cancer, chronic lung disease, overweight; working with manufacturers/designers of wearable sensors
	Low cost/disposable devices
	Provision of loan devices by clinical service providers
	Lower cost devices, open-source data access and software. that is usable!
**Poor health and/or digital literacy**	Improve understanding of digital devices by older people
	Wait for more digitally connected pts to age!
	Education and communication with support networks i.e. family or friends
	Increased support from clinical staff, both verbal and written
	Easy to use devices plus step-by-step instructions on use. Combined with regular monitoring of the data so the researcher can determine early if the device isn't being used/used correctly
	Support for user by clinical staff or relative/carer

Question 13: (optional) What are some strategies to address the other categories of patient-related barriers?

	Have a structured approach to prescription of the device (e.g. clinician needs to be aware of the recovery stage of a person with an eating disorder and if the device might trigger behaviours not conducive with recovery [i.e. calorie counting])

	All categories relate to pt engagement (tied to clinician skill in engaging pt) in managing their own health - even re resource barriers - if pt engaged, will advocate for / find resources, will overcome other clinical barriers
	Education/ opportunity to demonstrate
	Having resources available would be really useful. I think this barrier really comes down to financial support and often lack of it.
	I don't understand why people with cognitive impairment, reduced physical capacity and limited mobility are included as being clinically unsuitable. I work with adults and children with cerebral palsy who use wheelchairs and they are interested in measuring their PA. Clinicians need to work collaboratively with their clients/patients to ensure they are educated as to why the monitor is useful. Goal setting and targets could be important for the patient.
	Some patients may initially be willing to wear a wearable but their motivation to wear it may change over time, becoming more reluctant. This is something to consider and depends on how long you are wanting the patient to wear the wearable for.
	Literacy - provide a short (important) orientation through training and a reference document.
	Resourcing can be improved by evidence of effectiveness of use
	Simple devices will help address lots of these issues. Also real life examples of how these devices have assisted in rehab previously. The ability of the devices to hide the data for the patient may also be very helpful, as some people will have poor self-regulation/ dysfunctional relationships with exercise.
	Wearable is a tool the other barriers are amenable to clinician Ax and change Mx strategies
	Get doctors to engage in use of wearables and sharing of information between doctor and patient
	Assess appropriateness, test drive over a shorter period of time, practice/feedback, be willing to cease practice if not appropriate
	Clinical unsuitability - for very slow walkers < 0.4m/sec for example where the fitbit Zip worn on the shoe does not detect steps, design an accelerometer that has an algorithm sensitive enough to detect steps in these very slow walkers

Clinician and interdisciplinary team-related barriers

Question 14: What do you think is the most significant category of clinician and interdisciplinary team-related barrier?

Clinician and interdisciplinary team-related barrier	Healthcare participants		Research participants		All Participants	
	Total Responses	% Responses	Total Responses	% Responses	Total Responses	% Responses
Time constraints and competing demands	10	52.63	12	50.00	22	51.16
Lack of skills	2	10.53	6	25.00	8	18.60
Individual clinician factors	4	21.05	3	12.50	7	16.28
Lack of procedures and support systems	3	15.79	3	12.50	6	13.95
Lack of greater team involvement	0	0.00	0	0.00	0	0.00

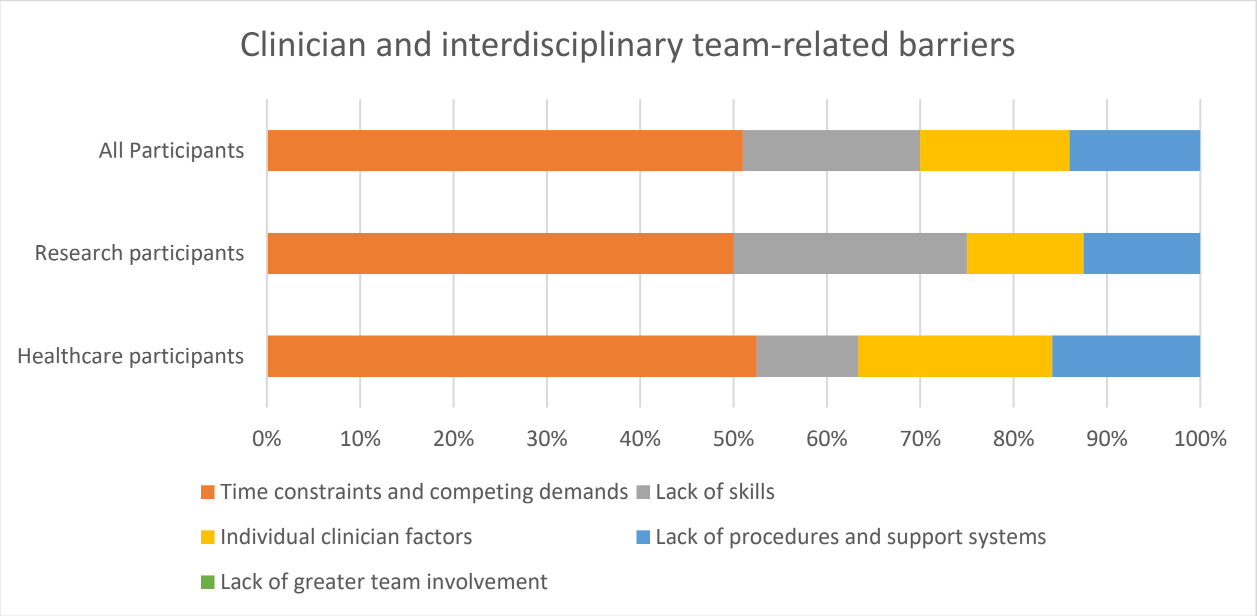

Question 15: (optional) What are some strategies to address this category of barrier?

**Time constraints and competing demands**	Pay them
	Time allocated for device management and data collection
	Altered funding model
	Improved resourcing of assistant/ technician workforce
	A straightforward and easy to understand device and interface, that is able to be used remotely, with easy upload of data and analysis. Also having easy and pre-set analyses available to save time if not able to customise. Ability to have a device set up prior to patient arrival, preferably from any device without needing to be physically attached to it. Simple "set up" guides.
	Needs to be tied to business plan, compensation, pay
	Closely followed by lack of procedures.
	To save clinician time, when multiple patients are wearing devices have an automatic daily upload to one central log in by a clinician for immediate feedback.
**Lack of skills**	Create opportunities for training of clinicians in the use of these devices and increase expectations about their standard use them in the clinical environment
	Education from researchers
	Part of this is due to the type of devices used - depending on the research question, using the simplest device possible to collect the data helps the patient/clinician/researcher. Consumer grade devices with their own apps mean data are usually pretty easy to interpret
	Written and verbal support (? training courses) for clinicians to improve their skills in this area.
	Training and digital champions that can help support the wider team.
	Training for clinicians in how to use and coach patients in use and understand reports and outputs
**Individual clinician factors**	Compliance strategies; Clinician education
	See above - if clinicians engage and are enthusiastic - will assist pt to engage
	Education!! Especially at an undergraduate level. Then find your experienced clinicians to act as champions. Not every physio will see the importance of PA and therefore measurement.
	Threshold level of utilization to make it normal, champions in the work place
**Lack of procedures and support systems**	Ensure the data from the patient can be uploaded to medical records or platforms like PhysiTrack. A clinician doesn't have time to seek out data, it should just be there for them to view
	Support team for research etc
	Development of a hot-line/help line to assist with details such as selection of wearable, and any issues accessing output

Question 16: (optional) What are some strategies to address the other categories of clinician and interdisciplinary team-related barriers?

	Increase capability, opportunity, and motivation to use the data produced by the device. This requires buy in - how will each data item enhance clinical care? How will it make the clinicians job easier?

	Related to team engagement and also support from wider organisation. can't separate one from the other.
	Education - at all levels: how to set goals for patients for PA; options for measuring; getting patients/consumers to advocate for using monitors; show and do with less experienced clinicians on how to use the software, the short-cuts, etc.
	I think we need to get the system working for us first, then others will get on board as they see the benefits. At the moment device use is such a jumble it puts others off seeing the benefit
	Top down leadership and creation of supportive policies and standard operating procedures.
	Clinical digital literacy
	Education, a "quick access" EBP guide, and maybe some documents that would assist in providing "business cases" to management to enable purchase and set up of these devices/ more time available to establish this aspect of service.
	Clinician paradigms are hard to break, need to find new acceptable paradigms
	Team work to teach others how to maximise use of wearable data
	Simple is better, straight forward, standardized protocols, policies that encourage use, etc.

Healthcare system-related barriers

Question 17: What do you think is the most significant category of healthcare system-related barrier?

Healthcare system-related barrier	Healthcare participants		Research participants		All Participants	
	Total Responses	% Responses	Total Responses	% Responses	Total Responses	% Responses
Lack of higher-level involvement	3	15.79	4	16.67	7	16.28
Ethical considerations	1	5.26	3	12.50	4	9.30
Lack of funding and resources	12	63.16	13	54.17	25	58.14
Lack of evidence	3	15.79	4	16.67	7	16.28

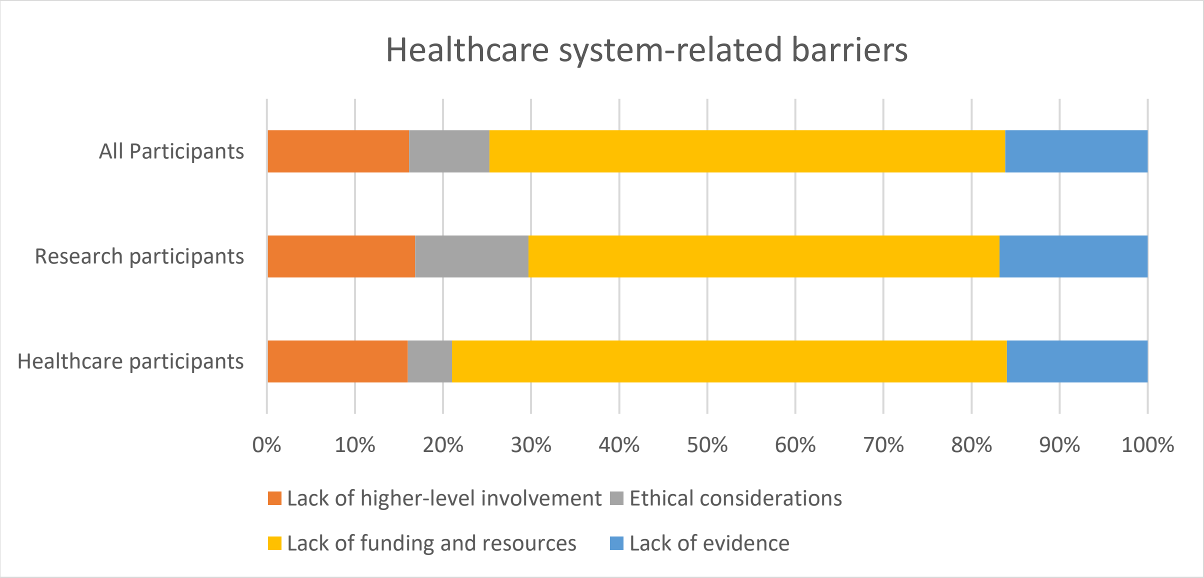

Question 18: (optional) What are some strategies to address this category of barrier?

**Lack of higher-level involvement**	Demonstrated benefits to health system operations and outcomes
	if this is addressed the other barriers will be too. i.e. if higher levels engage - will provide more funding and support. Engagement of high level organisation needs to backed up by decent research, also can be pt/clinician driven
	Need high-level champion in government (minister, CMO, CHO)
	Get the system working for enthusiastic users, then put a case forward for funding/support systems
**Ethical considerations**	
**Lack of funding and resources**	More advocacy at government levels. So much of health care is about containing costs that the need for an investment, especially in rehabilitation, is being lost
	Grants, encourage students to assist
	Grants esp if supported by consumers who see the value of measuring PA
	Evidence is needed to justify the use of wearables in clinical settings. This will help the case for increased funding for resources. Patient advocates are also useful.
	More funding
	Support for initiatives to provide proof of concept.
	Maybe some documents that would assist in providing "business cases" to management to enable purchase and set up of these devices/ funding for more time available to establish this aspect of service.
	Seed funding of wearables with high utility to demonstrate value and potential efficiencies to be gained
	Commonwealth to fund use of wearables and use of data
	Policies to priortize, mandate
**Lack of evidence**	Gather more evidence of effectiveness; research projects to prove health benefit outcomes

Question 19: (optional) What are some strategies to address the other categories of healthcare system-related barriers?

	As above for clinicians. (Increase capability, opportunity and motivation to use the data produced by the device. This requires buy in - how will each data item enhance clinical care? How will it make the clinicians job easier?)
	Prove the worth - show the policy makers and funders what information can be collected and positive ways it can be used. Demonstrate cost savings by moving people out of hospital earlier or preventing re-admissions.
	Evidence base will lead to better executive buy in and resourcing
	I think as evidence and research in this area increase, the other constraints will get easier to navigate and work through.
	or the "system" it is all about health economics
	get medicine to lead the charge and also have review and use of wearable data as a funding item in medicare
	the others are manageable if you change the system through policies first
