# Supplementary material for: Establishing a Consensus-Based Framework for the Use of Wearable Activity Trackers in Health Care: Delphi Study
Source: JMIR Mhealth Uhealth. 2024 Aug 23;12:e55254. doi: 10.2196/55254 (PMC11380062; doi:10.2196/55254)
Supplement: Multimedia Appendix 5 [file mhealth_v12i1e55254_app5.pdf]

<u>Round 4 Results</u>

38 total responses at close of survey = 65.5% response rate
Participants grouped into one of two categories based on professional background: Primary health system (including clinicians, health-system administration); Primarily research. Results displayed for each category, and total sample.

Item ratings were categorised as: ‘not useful’ (1-3), ‘neutral’ (4-6), and ‘very useful’ (7-9), and; ‘not clear and appropriate’ (1-3), ‘neutral’ (4-6), and ‘very clear and appropriate’ (7-9)

Consensus for an item as ‘very useful’ or ‘very clear and appropriate’ was defined as ≥75% of participants rating ≥7, and ≤15% of participants rating ≤3. Items were considered ‘somewhat useful’ or ‘somewhat clear and appropriate’ when between 50-75% rated an item as ‘very useful’ or ‘very clear and appropriate’ (score ≥7), and ‘not useful’ or ‘not clear and appropriate’ when less <50% rated it as very useful’ or ‘very clear and appropriate’.

Item 1 of 12.

Why?

Why will wearable activity monitors be used in the service?

Identify the purpose(s) and goal(s).

Explanation: The rationale, goals, and theory underpinning the use of wearable activity monitors should be clear. For example, reasons for using wearable activity monitors in healthcare settings may be to assess and monitor daily activities (i.e. steps, minutes of sleep, sedentary time, light or moderate-to-vigorous activity, and/or timing of activities such as bed time and wake time); intervene on daily activities (typically, steps, MVPA, sedentary behaviour or sleep); and potentially, monitor particular physiological parameters (e.g. heart rate during exercise sessions). Describing any underlying theories or behaviour change techniques will assist in planning procedures for the wearable activity monitors' use (for example, physical activity goal setting, prompts to move to break up sedentary behaviour, or feedback on sleep duration) [1]. Outlining the purpose(s) and goal(s) for using wearables will help differentiate essential elements from those that are incidental or optional.

	Primarily Health System					Primarily Research					All Participants				
	Likert Scale			Total Responses	% of responses = 3	Likert Scale			Total Responses	% of responses = 3	Likert Scale			Total Responses	% of responses = 3
	1-3	4-6	7-9			1-3	4-6	7-9			1-3	4-6	7-9		
This item is useful	1	1	16	18	88.89	0	1	18	19*	94.74	1	2	34	37	91.89
This explanation is clear and appropriate	1	0	17	18	94.44	0	0	20	20	100.00	1	0	37	38	97.37
*=1 response missing															

Do you have any comments on, or suggestions for improving the wording of this item?	This likely relates to all items, but having a decision tree / breaking the explanation into the options they want (which will then inform the device required) would practically be very helpful. The current wording helps the clinician think about what is needed, but not necessarily what to use.
	It may be worth mentioning the clarity of the timeframe of the monitoring period
	I can apply this item in a paediatric tertiary hospital, with a range of patients.
	I'm not at all sure most clinicians would have a very clear idea about the potential uses of wearables.
	I think patients need to understand how these data will be integrated with other datasets and how this will improve their experience of healthcare
	more straightforward text in the explanation e.g. take out 'for example', 'typically', 'potentially'. Put the last sentence as the second sentence.
Do you have any comments on, or suggestions for improving the wording of this explanation?	remove parentheses from para and consistency of "e.g." or "for example", if using i.e. suggest use e.g.
	Use of bullet points for ease of reading and perhaps add a final bullet such as, any other purpose that the results of the device will have a direct impact on care etc.
	it is quite long. could bullet points be used?
	It is too lengthy
	You could add something about the value of devices to motivate patients to engage in activity, engage with other users socially, and learn more about their own patterns of activity?

Item 2 of 12

Where?

In what setting/clinical context will you be using wearable activity monitors?

Identify the setting and features of the health service, including location(s), nature and volume of patient contact, and relevant infrastructure (e.g. suitable internet connection, spaces for patients to walk safely). Identify if previous efforts have been made to implement wearable activity monitors in the service.

Explanation: Wearable activity monitors may be valuable in a wide range of healthcare settings and services [2]. Why and how wearable activity monitors are used will vary depending on the needs of different settings and services. For example, in settings with limited face-to-face contact between patients and clinicians, wearables can provide remote insights into patient activity for clinicians, and provide additional motivation and feedback for the patient [3]. However, with less direct clinician involvement, the patients and their family members may be required to take more responsibility for the wearable (e.g. charging it, and fitting it after showering). Outlining the characteristics of the setting will assist in the selection of suitable wearable activity monitors, and the development of appropriate procedures for successful use. Additionally, identifying if and how wearable activity monitors have been used in the health service before (and what did and didn't work) may help with the planning and conduct of the current project.

	Primarily Health System					Primarily Research					All Participants				
	Likert Scale			Total Responses	% of responses = 3	Likert Scale			Total Responses	% of responses = 3	Likert Scale			Total Responses	% of responses = 3
	1-3	4-6	7-9			1-3	4-6	7-9			1-3	4-6	7-9		
This item is useful	1	2	15	18	83.33	0	2	18	20	90.00	1	4	33	38	86.84
This explanation is clear and appropriate	1	1	16	18	88.89	0	1	19	20	95.00	1	2	35	38	92.11

Do you have any comments on, or suggestions for improving the wording of this item?	a bit wordy
	No suggestions this reads very well and is clear
	Again, I think you need to point out concretely what wearables can be used for: monitoring PA, sleep, etc., and how they can provide a measure of the success of treatment.
	It doesn't say anything beyond the obvious. As part of the Delphi process we must be able to get closer to best practice guidelines
	Both prompt and explanation are wordy. Can these be simplified, particularly the prompt? The last part of the explanation (previous attempts and outcomes) might be unnecessary - surely what did and didn't work would have been done as quality improvement or research outcomes...?
Do you have any comments on, or suggestions for improving the wording of this explanation?	remove first set of parenthesis in para, keep second
	It is too lengthy
	Prompt: Identify the health service setting - location, nature of patient contact, relevant infrastructure. Explanation: Wearable activity monitors may be valuable, depending on the needs of different settings and services [2]. In settings with limited face-to-face contact, wearables can provide patient activity for clinicians, and motivation and feedback for patients [3]. The patients and family members may need to take responsibility for the wearable (e.g. charging it, and fitting it after showering).

Item 3 of 12

Who? (patients)

Who are the patients that wearable activity monitors will be used with?

Identify the patients (clinical group(s), characteristics, age range, mobility/ambulation, socioeconomic factors, typical goals etc.), what they will be required to do (e.g. monitoring activity, charging device, syncing data), and what they might need (e.g. information and instructions).

Explanation: Wearable activity monitors may be useful for measuring and intervening on physical activity for various patient populations [4]. In addition to identifying the clinical population(s) of interest, outlining typical patient characteristics can help to develop appropriate procedures for use, and plan for any support that patients may require. Patients may be required to interact with the device (e.g. to set goals and monitor activity) and carry out some minor responsibilities (such as charging and syncing). Devices may be complex for patients to manage, and they will likely require guidance and informational resources [5]. Patient factors to consider can include age, socioeconomic factors (availability of resources, support and proximity to care), mobility and ability to ambulate safely and independently, cognitive capacity to engage with interventions and manage devices, and typical goals. Identifying what patients will be required to do can highlight what specific information or instructions will be necessary for them [*see item 12 'Resources'*].

	Primarily Health System					Primarily Research					All Participants				
	Likert Scale			Total Responses	% of responses = 3	Likert Scale			Total Responses	% of responses = 3	Likert Scale			Total Responses	% of responses = 3
	1-3	4-6	7-9			1-3	4-6	7-9			1-3	4-6	7-9		
This item is useful	0	1	17	18	94.44	1	0	19	20	95.00	1	1	36	38	94.74
This explanation is clear and appropriate	1	1	16	18	88.89	0	1	19	20	95.00	1	2	35	38	92.11

Do you have any comments on, or suggestions for improving the wording of this item?	Really important to asses how tech savvy the patients and staff are. Patients can have smartphones for example, but may not know how to download and access an app. Staff may also not know how to guide a patient to set-up an app on their smartphone, for example
	Nil suggestions
	who are the patients with whom wearable activity monitors will be used
	The wording in the first brackets about the patients is very wide ranging. Typical goals doesn't sit that well with the other demographic type features
	As previously, simplify the wording especially for the prompt e.g., who? patient demographics (also avoiding ending the sentence with a preposition, if there are any grammar sticklers reading...). I'm not sure of the need to 'label' a patient group - the patients know who they are and what they need to do; they need to know when and how to use the device.
Do you have any comments on, or suggestions for improving the wording of this explanation?	Separate para for patient factors
	mobility may include a minimum gait speed if a minimum gait speed is required for an accelerometer to count a step
	Perhaps the inclusion of items relating to the assessment of technological capability and/or resources. Some population groups will not have access to required companion devices and/or internet, so differences in deployment may be required. Apologies if covered in a subsequent point.
	Patients also require sufficient dexterity
	Refer to sleep and sitting as well as PA.
	It is too lengthy

Item 4 of 12

Who? (providers)

Who are the clinicians leading the use of wearable activity monitors in the service?

Identify the lead clinicians' role and scope, what they will be required to do (e.g. assist set-up, review data, facilitate goal setting, promote activity monitoring and engagement), and what they might need (e.g. instructions and training, dedicated time).

Explanation: Healthcare professionals from varied backgrounds are increasingly using wearable activity monitors in different types of services [2]. Different professions will vary in their scope and the type of services provided, which will influence the purpose(s) and procedures for how devices are used in different contexts. In larger interdisciplinary teams, some professions may be primarily driving the use of wearable activity monitors and delivering services that involve wearables, while other clinicians may have a smaller role. Deciding which clinician(s) are leading the use of wearables and outlining what their role and responsibilities are can help with meeting the purpose(s) for use, and provide clarity for users and consistency within teams. Also consider if a knowledge and/or skill gap exists for these clinicians in fulfilling their role, which may need to be addressed to support successful use [*see item 12 'Resources'*].

	Primarily Health System					Primarily Research					All Participants				
	Likert Scale			Total Responses	% of responses = 3	Likert Scale			Total Responses	% of responses = 3	Likert Scale			Total Responses	% of responses = 3
	1-3	4-6	7-9			1-3	4-6	7-9			1-3	4-6	7-9		
This item is useful	0	4	14	18	77.78	0	2	18	20	90.00	0	6	32	38	84.21
This explanation is clear and appropriate	0	4	14	18	77.78	0	2	18	20	90.00	0	6	32	38	84.21

Do you have any comments on, or suggestions for improving the wording of this item?	Also consider if a knowledge and/or skill gap exists for these clinicians in fulfilling their role, which may need to be addressed to support successful use [see item 12 'Resources']. Modified to; Clinicians should consider if a knowledge and/or skills gap.....for them....
	Having a single profession overseeing would also make it clear for the participants who they should be contacting for troubleshooting and support if required during the process
	Probably need to refer to technical support. It is unlikely a clinician will be providing this.
	simplify wording. Prompt - avoid full sentences, use key words only. Explanation - simplify.
Do you have any comments on, or suggestions for improving the wording of this explanation?	May be difficult to clearly define lead clinician if several are keen to use the data for sl. different purposes
	It is too lengthy

Item 5 of 12

Who? (additional)

Who else is involved in supporting patients and services using wearable activity monitors?

Identify any additional personnel, their profession (e.g. nurse, administration) or relationship (e.g. carer/family), what they will be required to do (e.g. check device is charged and worn, provide encouragement, keep track of loan devices), and what they might need (e.g. information and instructions).

Explanation: Consider the members of the multi-disciplinary team (e.g. nursing staff, doctors, allied health), or patient carers and relatives that may facilitate wearable activity monitor use. Additional personnel can support use by checking that devices are charged and worn correctly and providing encouragement to patients. The needs of the patient and their capacity to independently manage the device and engage with care should be considered when enlisting the assistance of additional personnel. For example, wearable activity monitors may provide valuable insights on activity in patients with cognitive impairments, but their ability to independently manage the devices and engage with interventions may be limited [2]. In such circumstances, involvement of a carer to remind patients to wear the device and provide encouragement may be a useful strategy to support successful use [6]. If additional personnel will be involved, consider if they require any specific information or resources to help support patients [*see item 12 'Resources'*].

	Primarily Health System					Primarily Research					All Participants				
	Likert Scale			Total Responses	% of responses = 3	Likert Scale			Total Responses	% of responses = 3	Likert Scale			Total Responses	% of responses = 3
	1-3	4-6	7-9			1-3	4-6	7-9			1-3	4-6	7-9		
This item is useful	1	2	14	17*	82.35	0	2	18	20	90.00	1	4	32	37*	86.49
This explanation is clear and appropriate	1	2	14	17*	82.35	0	1	19	20	95.00	1	3	33	37*	89.19
*=1 response missing															

Do you have any comments on, or suggestions for improving the wording of this item?	no comments on wording, but a general comment. this is a good question as in health care settings it would be quite hard to rely on other professions to help clients with wearables, the training would be hard to implement. this questions helps to identify that the researcher would likely "be on their own"
	I don't find this additional question adds much to the first four.
	Take out the examples in the prompt; simplify the wording again (sorry). Explanation - probably don't need to list the M-D team members, take out 'for example', less essay and more factual?
Do you have any comments on, or suggestions for improving the wording of this explanation?	agree, keeping track of loan device- who is responsible, particularly on patient discharge from a IP hospital bed
	It is too lengthy

Item 6 of 12

What? (metrics)

What are the metrics of interest? (e.g. steps, daily minutes of physical activity, daily minutes of sedentary behaviour)

Consider relevance to the purpose(s) and population, and accuracy for the population (including wear location).

Explanation: Wearables collect a range of metrics, which vary between models. The key metrics of interest will be influenced by the purpose(s) and populations the wearable activity monitors will be used with. Identifying the key metrics of interest can guide device selection. Common and relevant metrics typically include:

- Daily step count
- Daily minutes of physical activity
- Daily minutes of sedentary time

  In some circumstances, other metrics, such as oxygen saturation and heart rate, may be of interest. The accuracy of specific metrics in different populations can vary across different makes and models of device [7, 8] as well as wear location on the body [9, 10]. Ideally, metrics should provide useful information to clinicians, be relevant to patients and their goals, and be sufficiently accurate and reliable in the populations they are being used with. For example, slow-walking older adult rehabilitation patients may be best suited to step metrics, as these would correspond to goals of reducing immobility and have demonstrated sufficient accuracy in these populations [11]. Whereas younger populations with increased physical capacity and goals of exercising at higher intensities may be well suited to MVPA minutes [12].

	Primarily Health System					Primarily Research					All Participants				
	Likert Scale			Total Responses	% of responses = 3	Likert Scale			Total Responses	% of responses = 3	Likert Scale			Total Responses	% of responses = 3
	1-3	4-6	7-9			1-3	4-6	7-9			1-3	4-6	7-9		
This item is useful	1	0	17	18	94.44	0	1	19	20	95.00	1	1	36	38	94.74
This explanation is clear and appropriate	1	0	17	18	94.44	0	2	18	20	90.00	1	2	35	38	92.11

Do you have any comments on, or suggestions for improving the wording of this item?	MVPA? should be clear to readers what this is without having to go search for it
	Add sleep. It's really important for recovery, and critical for mental health.
	The focus on accelerometer based wearables here is way to much. What about HR monitors, GPS devices, skin impedance, continuous blood sugar monitoring, etc…
	Same as all previous - simplify the language and volume of content. Explanation - perhaps just a list of possible metrics as a checklist?
Do you have any comments on, or suggestions for improving the wording of this explanation?	I question the inclusion of sedentary time within this description. Whilst I fully agree it is useful, many wearables still only really provide activity metrics rather than inactivity metrics. I would also suggest including something to the effect of: - clinicians should be aware that metrics between devices may not align exactly and that this should be taken into account when comparing back to studies in patient groups / between current patients using different devices.
	Think need to look again at references in support of the statement that step counts are accurate on slow walking populations, or reword this example as this is not always the case depending on the device and wear location, and inpatient vs outpatients may differ. Ie this is not always true for the activpal
	More information on selection
	I'd add that other relevant metrics can include SpO2 etc (whatever you have been told by other participants) in the dot points as this can help draw the eye to the most relevant information for the clinician
	It is somewhat lengthy

Item 7 of 12

What? (device characteristics)

What device(s) will be used, and what are the available characteristics?

Consider if the device and its characteristics support the purpose(s), will meet users' needs, and the practical considerations for ongoing use.

Explanation: Characteristics to consider will typically relate to:

- Wearing the device (bodily wear site, water resistance, comfort, ease of cleaning)
- Charging the device (battery life, frequency of charging (daily vs weekly vs yearly), time taken to charge)
- The device interface (feedback that is easy to understand, feedback provided on device screen vs. an application?)
- Ease of set up and navigation
- Frequency and interpretability of feedback provided
- Any additional features (personalized goal setting, additional smart devices/applications that link to the wearable device)

Consider which specific characteristics meet the needs of the service and users. For example, devices that are worn on the wrist, simple to use, comfortable, and have attractive and discreet designs may be advantageous for patient adherence and engagement [13]. Certain characteristics may also provide additional benefits for activity interventions that the devices are being used for. For example, devices that provide real-time feedback and prompts to be active can be motivating for patients in increasing their physical activity [14].

	Primarily Health System					Primarily Research					All Participants				
	Likert Scale			Total Responses	% of responses = 3	Likert Scale			Total Responses	% of responses = 3	Likert Scale			Total Responses	% of responses = 3
	1-3	4-6	7-9			1-3	4-6	7-9			1-3	4-6	7-9		
This item is useful	0	1	17	18	94.44	1	2	17	20	85.00	1	3	34	38	89.47
This explanation is clear and appropriate	0	1	17	18	94.44	1	1	18	20	90.00	1	2	35	38	92.11

Do you have any comments on, or suggestions for improving the wording of this item?	What device needs to include validity indices related to accuracy, precision, bias. At a minimum published MAPE values obtained under controlled conditions. These are now available and should be routinely reviewed and/or devices tested to produce MAPE values that can be compared to published values. Kids and adults MAPE catalogs with expected values are available to help with interpretation (see Tudor-Locke, IJNBPA). Focusing on just the characteristics of devices with no concern for performance is problematic.
	I guess what clinicians really need is a go-to site where the characteristics of different devices are clearly laid out. This is at a level of only moderately useful generality.
	surely the device chosen will meet the requirements, otherwise why would it have been chosen?
Do you have any comments on, or suggestions for improving the wording of this explanation?	Could include, device requirements e.g. smart phone / internet connected device, version of operating system etc. Most devices are cross platform but there are still some patients that do not have the most up to date software, stopping them from using the required apps.
	Perhaps mention wear-site suited to populations where wrist-wear is difficult, e.g. healthcare workers who may not be able to wear on the wrist
	Not just prompts to be active, but to stand up (ire break up sedentary behaviour)
	I didn't understand what interpretability of feedback meant
	It is somewhat lengthy

Item 8 of 12

How? (procedures)

How will wearable activity monitors be used in the service?

Outline the procedures for using wearable activity monitors and what needs to be done to meet the purpose(s) of use, support users, use devices as intended, and care for and maintain devices.

Explanation: Clear procedures and planning can support successful implementation and ongoing use. Appropriate procedures can enable consistency in how devices are used, and may help to circumvent potential difficulties that may arise (such as with charging and syncing, correct wear or device loss) [2]. When planning and developing procedures for use, consider:

- How devices will be set up for patients
- How metrics and data outputs will be used and documented
- How activity intervention and promotion will be addressed (including behaviour change techniques)
- Strategies for fidelity and adherence
- How devices will be cared for ongoing
- How devices will be distributed and managed.

Procedures should correspond to the purpose(s) of use, and consider the needs of the users involved [15]. This may be achieved though undertaking a collaborative approach that involves relevant users (e.g. all relevant clinicians, patients, carers, administration) when planning and developing procedures. Pilot testing the device and the procedures prior to implementation can help to identify and address any potential problems.

	Primarily Health System					Primarily Research					All Participants				
	Likert Scale			Total Responses	% of responses = 3	Likert Scale			Total Responses	% of responses = 3	Likert Scale			Total Responses	% of responses = 3
	1-3	4-6	7-9			1-3	4-6	7-9			1-3	4-6	7-9		
This item is useful	1	1	16	18	88.89	0	1	19	20	95.00	1	2	35	38	92.11
This explanation is clear and appropriate	0	2	16	18	88.89	0	1	19	20	95.00	0	3	35	38	92.11

Do you have any comments on, or suggestions for improving the wording of this item?	fidelity? accuracy/ precision; Fidelity has more than one meaning
	consider whether data can be shared with electronic medical record systems. Also consider data security and managment
	Pilot testing will be a great addition to the process to troubleshoot
	Q2 uses the words setting/clinical context (vs service), might be good to standardise the terminology
	there is a risk that this item will get lost with the other following items about how data is captured etc. - you could probably roll some of these together.
	Prompt: "procedures to meet the intended purpose, support users, care for and maintain devices."
Do you have any comments on, or suggestions for improving the wording of this explanation?	'How devices will be cared for ongoing' could be clearer Perhaps, 'How ongoing care (e.g., cleaning and charging) of devices will be managed'
	Are the procedures for clinicians or users?
	A very full explanation is needed for this and how it intersects with some others eg metrics
	Explanation - just the dot points?

Item 9 of 12

How? (data access)

How will data be accessed and managed?

Outline the method of data access and management, software and applications that will be used, the frequency/time points that data will be reviewed, and by whom.

Explanation: Methods for data access and handling should be compatible with data handling systems in the service [16]. The methods for accessing and handling data can vary in complexity and cost. Data access may be as simple as reading and recording outputs from the device interface and/or accompanying applications, or it may be more complex and involve downloading raw data sets and use of third-party software to conduct comprehensive analyses. How data will be accessed and used will be influenced by the purpose(s) of use and the resources (human and cost) available. Consider the available software or applications, and the feasibility of acquiring specific software and technology (such as tablets) if required. Procedures for data access will involve reviewing the data at different time points and frequencies, and by specific users and personnel. When outlining data access procedures for successful and consistent use, consider the method of data access, and who will be responsible for accessing and handling data.

	Primarily Health System					Primarily Research					All Participants				
	Likert Scale			Total Responses	% of responses = 3	Likert Scale			Total Responses	% of responses = 3	Likert Scale			Total Responses	% of responses = 3
	1-3	4-6	7-9			1-3	4-6	7-9			1-3	4-6	7-9		
This item is useful	1	2	15	18	83.33	0	2	18	20	90.00	1	4	33	38	86.84
This explanation is clear and appropriate	1	4	13	18	72.22	0	2	18	20	90.00	1	6	31	38	81.58

Do you have any comments on, or suggestions for improving the wording of this item?	See comments on previous question
	Doesn't address potential for derived variables, using direct output and manipulating it for specific needs. For example, some devices now only provide raw signals that need to be manipulated. Some provide some variables that need to be summed and/or averaged to get at the desired end point. Derived variables need some direction too so they are standardized so people aren't performing different manipulations and calling them the same things.
	Data security, cloud security and approvals, privacy are all issues that jump to my mind
	I'm not sure this is very useful at this level of generality. It would seem obvious, for example, that incoming data would need to be compatible with the data handling procedures of the service.
	How (data access) is contrived - why not just Data Access
	We should be moving to cloud reporting and automated analysis of these data
	as before this and the previous item are two sides of the same coin. If the person isn't wearing the device then data will not be captured. acknowledge that even if everything works well still need to have a way of retrieving the data and whether its in real time or downloads. Are there key issues in these questions where if the step is not reached then there is no further work done?
	Prompt: "Outline data access and management, software and applications, frequency/time points for data review, person responsible." Explanation - as previously, suggest simplifying the wording so it reads factually rather than having the additional linking phrases like an essay
Do you have any comments on, or suggestions for improving the wording of this explanation?	Importantly, who will be responsible for setting up and managing the accounts. Will the patient set up their own account (which could prevent clinicians from viewing data from cloud based systems), or will all patients be given a study ID account that they are given access to?
	Maybe highlight as a separate sentence the costs involved, especially for clinical time and software required. Potentially, in data management.
	It took a few reads to get the meaning. I understand the message, but need to understand it on the first read. Particularly the second half of the explanation

	As for 26 (Data security, cloud security and approvals, privacy are all issues that jump to my mind)
	If the clinician intents to extract the data rather than reviewing the data within the device's software, they should consider the granularity of the data they are collecting - minute level data sounds great right up until you're trying to extract it and dealing with thousands of rows of data for each person every day. Device accuracy and noise becomes an issue with very granular data too...
	Data management should be regulated according to existing institutional practice, not bespoke for the instance

Item 10 of 12

When and how much?

When will measures be taken?

When will devices be provided to patients, and how long do they need to wear them for?

Identify timepoints for providing devices and ceasing use, the frequency/time points that data will be reviewed, and how long patients need to wear devices for valid measures.

Explanation: A baseline and key timepoints for assessing outcomes with wearable devices will need to be established. The baseline may be at commencement or a specific timepoint in treatment (e.g. at intake into the service), or when a specific therapy milestone is reached (e.g. ambulate independently). Consider when the device will be provided to patients, as this may need to be prior to the formal baseline, to allow for sufficient data to be collected to form a baseline measure (e.g. the week before). The device will need to be worn for enough time during a 24-hour period to provide valid measures of activity. A minimum time of 10 waking-hours per day is generally considered suitable to capture daily physical activity measures [17]. If devices are provided to patients by the service on loan (as opposed to patients obtaining ownership of their own device), the timepoint or milestone that use will be ceased should be specified in advance to prevent device loss.

	Primarily Health System					Primarily Research					All Participants				
	Likert Scale			Total Responses	% of responses = 3	Likert Scale			Total Responses	% of responses = 3	Likert Scale			Total Responses	% of responses = 3
	1-3	4-6	7-9			1-3	4-6	7-9			1-3	4-6	7-9		
This item is useful	1	1	16	18	88.89	0	1	19	20	95.00	1	2	35	38	92.11
This explanation is clear and appropriate	1	0	17	18	94.44	0	1	19	20	95.00	1	1	36	38	94.74

Do you have any comments on, or suggestions for improving the wording of this item?	Would it be also worth considering the days on which the devices are worn, for example 10Hrs on a weekend day may be very different to a week day?
	Again it is contrived - call It Usage or Implementation
	Your definition of wearable device seems to have been purely focused on accelerometers
	This is the third part of the conversation about data, how it is captured and what it means. This may lead people down a garden path. Could the data items be grouped in some way which show the elements that need to be considered?
	frequency, intensity and duration
Do you have any comments on, or suggestions for improving the wording of this explanation?	for loan devices - consider recovery of device at least one day prior to their discharge date
	Perhaps include a comment regarding what to do about charging: how often etc and best time to do so? This could be put within point 8, but specifically relates to the 10 hour wear time comment.
	Encourage clinicians to think like researchers and write a methodology, being aware of the characteristics of the patients.
	Should there be a recommended number of days as well as minimum number of hours per 24 h period?
	This is a complex item with many possible variatiins and needs to be backed up with a data design plan, not just a list of considerations
	This may only be a researcher problem but reactivity can influence participants who use devices for only a short period - the novelty of the device can mean participants are more active than usual/than they will be after a few weeks or after the novelty has worn off. Activity captured by the device may not be representative of their usual activity simply because having the device on encourages them to be more active than they would otherwise

Item 11 of 12

Adaptations and modifying

Will the procedures and use of devices need to be adapted in some circumstances?

Consider if/how device use will be adapted or modified for different patients or circumstances. Identify the modification and justification (e.g. different bodily wear site in very slow walkers).

Explanation: In any given setting, individual patient circumstances and presentations will vary. Modifications and adaptations to standard procedures may be required in some circumstances. While not all circumstances can be foreseen, planning for possible and likely modifications can support patients and clinicians in carrying out alternate procedures when necessary. For example, a mixed rehabilitation service may see various patients with different ambulatory capacity. In such circumstances the wear site may vary for different clinical groups such as very slow walking and/or post-stroke patients, for whom the ankle or shoe is a more suitable wear site for more accurate measures [11, 18]. Other factors to consider might include availability of carers and support, cognition and capacity for managing devices and engaging with interventions, health and digital literacy, different capacities for activity, ability to safely and independently ambulate.

	Primarily Health System					Primarily Research					All Participants				
	Likert Scale			Total Responses	% of responses = 3	Likert Scale			Total Responses	% of responses = 3	Likert Scale			Total Responses	% of responses = 3
	1-3	4-6	7-9			1-3	4-6	7-9			1-3	4-6	7-9		
This item is useful	1	1	16	18	88.89	0	4	16	20	80.00	1	5	32	38	84.21
This explanation is clear and appropriate	1	2	15	18	83.33	0	2	18	20	90.00	1	4	33	38	86.84

Do you have any comments on, or suggestions for improving the wording of this item?	This section would really come down to the individual circumstances, would justifying the decisions and or changes in a transparent manner be useful here?
	Prompt: "Consider adaptation or modification circumstances and justification"
Do you have any comments on, or suggestions for improving the wording of this explanation?	Other circumstances could include blinded baseline periods. Can devices be blinded or can the app data be removed for true baseline assessments?
	It is too lengthy
	Explanation - it's very wordy again. the content is there but can you condense by referring to the first 10 items (why, who etc.) with key points such as position, care of wearable etc.?

Item 12 of 12

Resources

What resources are needed to support users?

Identify what the different users involved need to support them in using wearable activity monitors in the service (e.g. information, training, software etc.).

Explanation: Supporting resources and instructions will be required for users (clinicians, patients, and additional personnel such as carers or administrative staff) [5, 19]. Resources can include information for users on why activity is important and why wearable activity monitors are being used (the rationale), as well as information and instructions on how to use the devices or any accompanying software (procedures). In addition to information resources, training and practical support may be required to address skill gaps. When developing resources for users, consider what information and training the different users will need, and consider collaborating with individuals who represent the different types of users to better understand their needs and preferences for resources.

	Primarily Health System					Primarily Research					All Participants				
	Likert Scale			Total Responses	% of responses = 3	Likert Scale			Total Responses	% of responses = 3	Likert Scale			Total Responses	% of responses = 3
	1-3	4-6	7-9			1-3	4-6	7-9			1-3	4-6	7-9		
This item is useful	1	0	17	18	94.44	0	2	18	20	90.00	1	2	35	38	92.11
This explanation is clear and appropriate	1	1	16	18	88.89	0	2	18	20	90.00	1	3	34	38	89.47

Do you have any comments on, or suggestions for improving the wording of this item?	No sure if this goes here or under some other point, but the concept of "reporting and interpretation" is conspicuously absent from the checklist. Are there reference/expected/normative values to help with the interpretation of outputs? What are these? What do the outputs mean? How will the data be reported? What is a standardized presentation? Who will receive the data? Participants? Clinicians? Researchers? What are they expected to do as a result of receiving this information?
	Prompt: "support with information, training, software etc."
Do you have any comments on, or suggestions for improving the wording of this explanation?	the wearable activity monitor also needs to also be clearly referred to in a general ward/Physio etc orientation document that relates to the ward (for example) that is using the devices - then refer to the specific resource
	highlight the costs involved and that the clinician/researcher should be generous when factoring time=money
	Something else about planning for device replacement and servicing and maintenance requirments, estimating/resourcing the number of required devices to suit the monitoring purpose (or make sure devices are available, especially when worn for prolonged periods or loaned time participants for return)
	It is too lengthy
	You often need ongoing Softwear support. E.g if you develop an app and your study runs for a couple of years then the ongoing app support is essential as with operating system upgrades your app will stop working
	Users should also be informed who will be able to see their data and who owns their data moving forward - can they download it, hide it, delete it themselves if they wish? If it's a commercial device - like apple or fitbit - who can access/use their data after their health data treatment is complete?
	Simplify (again, sorry). Separate sentence 2 into 2 sentences. Take out the 'in addition to'...

Overall feedback

Do you have any further comments or feedback on the checklist and explanations?	thank you
	I think the checklist covers all the key areas and the explanations are excellent.
	As indicated, I think all the items are useful and relevant. But I do think it will be difficult for clinicians to answer the questions without fairly extensive knowledge. As flagged on the first point, it will be important (I think) to develop a decision making tool to help Clinicians choose the right approach / or give them options for this (with associated cost decisions). Perhaps at the start of the tool they can indicate what devices they have available to them.
	No, all very clear and well put together.
	well done, this seems really thorough. nothing to add.
	Great idea compiling the checklist. Look forward to the final version. Thanks
	Well done, coming together into a comprehensive document
	I didn't see anything re skin care which is important for devices that are attached to the skin such as activPAL. Sorry if I missed this. Skincare is an important factor and can limit data collection, and impact the relationship with the patient if not addressed
	The explanations are a a high level of generality. Most are pretty obvious. I was expecting some more concrete suggestions.
	I'd add a one-page tick-box list in front of the checklist that is broken up into the 12 items and where each item has up to 5 things the clinician must tick or cross before reaching the end of the checklist. More to help them to record what they have done and to easily and quickly apply the tool
	Great work!!
	I believe you need shorter explanations and then provide a very comprehensive expanded version backing that, with case studies and references. At yhe moment they sound like you try to teach the reader the high level concepts needed for each item but it is neither intuitive, nor comprehensive
	The questions are good, but would a flow chart or visual diagram be of assistance to represent the issues in some other way - to show how the decisions need to be taken? a visual may act as a summary of what the issues are prior to the questionnaire being undertaken.
	There has been a great deal of thought and work gone into this version, with many current references to support the suggestions - well done! Excellent content throughout. However, although the wording is written like an (excellent) essay, it would benefit from being more direct throughout. Currently the salient points are missed and key information is lost. The item titles, while clever, are repeated which loses impact. The keyword for each item is in brackets and could be the item title.

References

1. Duncan, M., B. Murawski, C.E. Short, A.L. Rebar, S. Schoeppe, S. Alley, C. Vandelanotte, and M. Kirwan, *Activity trackers implement different behavior change techniques for activity, sleep, and sedentary behaviors.* Interactive journal of medical research, 2017. **6**(2): p. e6685.
2. Maher, C., K. Szeto, and J. Arnold, *The use of accelerometer-based wearable activity monitors in clinical settings: current practice, barriers, enablers, and future opportunities.* BMC Health Services Research, 2021. **21**(1): p. 1064.
3. Wu, H.S., R. Gal, N.C. van Sleeuwen, A.C. Brombacher, W.A. Ijsselsteijn, A.M. May, and E.M. Monninkhof, *Breast Cancer Survivors' Experiences With an Activity Tracker Integrated Into a Supervised Exercise Program: Qualitative Study.* JMIR Mhealth Uhealth, 2019. **7**(2): p. e10820.
4. Wu, M. and J. Luo, *Wearable technology applications in healthcare: a literature review.* Online J. Nurs. Inform, 2019. **23**(3).
5. Ummels, D., E. Beekman, A. Moser, S.M. Braun, and A.J. Beurskens, *Patients' experiences with commercially available activity trackers embedded in physiotherapy treatment: a qualitative study.* Disability and Rehabilitation, 2020. **42**(23): p. 3284-3292.
6. Farina, N., G. Sherlock, S. Thomas, R.G. Lowry, and S. Banerjee, *Acceptability and feasibility of wearing activity monitors in community-dwelling older adults with dementia.* International Journal of Geriatric Psychiatry, 2019. **34**(4): p. 617-624.
7. Straiton, N., M. Alharbi, A. Bauman, L. Neubeck, J. Gullick, R. Bhindi, and R. Gallagher, *The validity and reliability of consumer-grade activity trackers in older, community-dwelling adults: A systematic review.* Maturitas, 2018. **112**: p. 85-93.
8. Evenson, K.R., M.M. Goto, and R.D. Furberg, *Systematic review of the validity and reliability of consumer-wearable activity trackers.* International Journal of Behavioral Nutrition and Physical Activity, 2015. **12**(1): p. 159.
9. Simpson, L.A., J.J. Eng, T.D. Klassen, S.B. Lim, D.R. Louie, B. Parappilly, B.M. Sakakibara, and D. Zbogar, *Capturing step counts at slow walking speeds in older adults: comparison of ankle and waist placement of measuring device.* Journal of rehabilitation medicine, 2015. **47**(9): p. 830-835.
10. Gaz, D.V., T.M. Rieck, N.W. Peterson, J.A. Ferguson, D.R. Schroeder, H.A. Dunfee, J.M. Henderzahs-Mason, and P.T. Hagen, *Determining the Validity and Accuracy of Multiple Activity-Tracking Devices in Controlled and Free-Walking Conditions.* American Journal of Health Promotion, 2018. **32**(8): p. 1671-1678.
11. Farmer, C., M.E.L. van den Berg, S. Vuu, and C.J. Barr, *A study of the accuracy of the Fitbit Zip in measuring steps both indoors and outdoors in a mixed rehabilitation population.* Clinical Rehabilitation, 2021. **36**(1): p. 125-132.
12. Ferguson, T., A.V. Rowlands, T. Olds, and C. Maher, *The validity of consumer-level, activity monitors in healthy adults worn in free-living conditions: a cross-sectional study.* International Journal of Behavioral Nutrition and Physical Activity, 2015. **12**(1): p. 42.
13. Henriksen, A., A.-S. Sand, T. Deraas, S. Grimsgaard, G. Hartvigsen, and L. Hopstock, *Succeeding with prolonged usage of consumer-based activity trackers in clinical studies: a mixed methods approach.* BMC Public Health, 2020. **20**(1): p. 1300.
14. Hardcastle, S.J., M. Galliott, B.M. Lynch, N.H. Nguyen, P.A. Cohen, G.R. Mohan, N.J. Johansen, and C. Saunders, *Acceptability and utility of, and preference for wearable activity trackers amongst non-metropolitan cancer survivors.* PLOS ONE, 2019. **13**(12): p. e0210039.
15. Keogh, A., K. Taraldsen, B. Caulfield, and B. Vereijken, *It's not about the capture, it's about what we can learn": a qualitative study of experts' opinions and experiences regarding the use of wearable sensors to measure gait and physical activity.* Journal of NeuroEngineering and Rehabilitation, 2021. **18**(1): p. 78.
16. Smuck, M., C.A. Odonkor, J.K. Wilt, N. Schmidt, and M.A. Swiernik, *The emerging clinical role of wearables: factors for successful implementation in healthcare.* npj Digital Medicine, 2021. **4**(1): p. 45.
17. Colley, R., S.C. Gorber, and M.S. Tremblay, *Quality control and data reduction procedures for accelerometry-derived measures of physical activity.* Health reports, 2010. **21**(1): p. 63.
18. Campos, C., V.G. DePaul, S. Knorr, J.S. Wong, A. Mansfield, and K.K. Patterson, *Validity of the ActiGraph activity monitor for individuals who walk slowly post-stroke.* Topics in Stroke Rehabilitation, 2018. **25**(4): p. 295-304.
19. van der Weegen, S., R. Verwey, M. Spreeuwenberg, H. Tange, T. van der Weijden, and L. de Witte, *It's LiFe! Mobile and Web-Based Monitoring and Feedback Tool Embedded in Primary Care Increases Physical Activity: A Cluster Randomized Controlled Trial.* J Med Internet Res, 2015. **17**(7): p. e184.
